# Thirty two port super wideband diversity antenna for indoor communications

**DOI:** 10.1038/s41598-024-76008-6

**Published:** 2024-10-23

**Authors:** Annal Joy J, Sandeep Kumar Palaniswamy, Sachin Kumar, Malathi Kanagasabai, Hyun Chul Choi, Kang Wook Kim

**Affiliations:** 1 Department of Electronics and Communication Engineering, Faculty of Engineering and Technology, SRM Institute of Science and Technology, Kattankulathur, 603203 India; 2 Department of Electronics and Communication Engineering, Galgotias College of Engineering and Technology, Greater Noida, 201310 India; 3 Department of Electronics and Communication Engineering, College of Engineering, Guindy, Anna University, Chennai, 600025 India; 4 School of Electronic and Electrical Engineering, Kyungpook National University, Daegu, 41566 Republic of Korea

**Keywords:** Engineering, Electrical and electronic engineering

## Abstract

This paper introduces a novel design featuring a thirty-two port diversity antenna with an elliptical shape, fed by an asymmetric coplanar waveguide (CPW). The antenna incorporates uneven meander lines, tailored for super-wideband (SWB) applications. The structure of the unit cell is of an elliptical patch with an elliptical slot, and it is connected to a rectangular stub and asymmetric meander line. The size of the single element is 22 mm × 20 mm, and it operates from 3 to 40 GHz. The bandwidth dimension ratio of the unit cell is 3911, bandwidth ratio is 13.33:1, and fractional bandwidth is 172.09%. The single element is developed into a 3-D thirty-two port diverse antenna, composed of a horizontal plane and four planes perpendicular to it. The diverse antenna has a peak gain of 12.5 dBi and an efficiency of 94%. The computed envelope correlation coefficient (ECC), as determined using S-parameters and far-field measurements, is less than 0.1. The attained diversity gains (DGs) of the developed MIMO antenna are above 9.9 dB in terms of both S-parameter and far-field computations. The obtained thirty-two port diverse antenna channel capacity loss (CCL) is below 0.25 bits/s/Hz. The mean effective gain (MEG) of the constructed thirty-two port antenna is below 2. To validate the appropriateness of the developed thirty-two port antenna for wireless indoor environment, an enclosure made of acrylonitrile butadiene styrene (ABS) is crafted and fabricated, and the characteristics of the proposed diverse antenna are investigated.

## Introduction

As wireless technology advances, there is an increasing demand for antennas that support a wide bandwidth, high data rates, and consume less power^1^. Although ultra-wideband (UWB) antennas provide wide bandwidth and high data rates, they are not preferable for emerging technologies due to their low radiation^2^. These days, fifth-generation (5G) systems^3^, Internet of Things (IoT)^4,5^, smart homes^2^, smart wearable devices^6^, and high-definition video streaming^7,8^, require antennas that provide improved data rates and ultra large bandwidth. The bandwidth ratio of the UWB antennas is ideally 3.4:1, whereas the bandwidth ratio of the super-wideband (SWB) antenna is larger than or equal to 10:1^9^. It covers a broad spectrum, including UWB, WLAN, Wi-MAX, ISM bands, IEEE 802.11, S, C, X, Ku, Ka, 5G NR, sub-6 GHz, and mm-wave bands^10^, whereas UWB antennas operate from 3.1 to 10 GHz^11–13^. Also, SWB antennas support 5G bands, making them better suited for IoT systems.

As the world moves towards smart agriculture, smart homes, smart vehicles, smart healthcare, smart cities, industrial automation, and restructured retail and inventory management, an IoT-enabled SWB antenna is required^14^. SWB antennas can be mounted in supermarkets, educational institutions, multi-story buildings, and locations with multiple users, where high data rate transmission, video streaming, and a large frequency range is required. Multipath fading is another important issue that must be addressed^15,16^. When the SWB antenna is placed in the said scenarios, there will be interference and loss due to scattering and deep signal penetration, which leads to multipath propagation. In these conditions, diversity antennas can be implemented to increase signal strength. Diversity enhances signal quality by attaining multiple identical signal information through different pathways. Integrating SWB wireless systems with multiple-input-multiple-output (MIMO) increases channel capacity and transmission power. Implementing a SWB MIMO antenna configuration enhances stability of the device, data transfer rate, reliability of the link, and throughput, resulting in enhanced system performance.

The need for polarisation flexibility has increased in recent years, resulting in the emergence of multiple antenna placement in 3-D configuration^17^. The 3-D antenna system offers more coverage as it emits radiation in both horizontal and vertical planes. The 3-D configuration can be used in devices where space is limited as it occupies the least amount of space while providing better performance. In^18^, a SWB MIMO antenna was developed, with electromagnetic band gap (EBG) structure for isolation. In^19^, an elliptical monopole MIMO antenna was constructed for SWB applications, but the single element was very large, measuring 33.5 mm × 33.5 mm. Therefore, the polarization freedom was reduced. A wideband diverse antenna was developed in^20^, but it covered a frequency range of 3 to 20 GHz only. In^21^, the reported SWB antenna had a wide bandwidth, it was large in size and had less polarisation flexibility, as it only had two ports in a 2-D configuration. In^22^, a self-complementary coplanar waveguide (CPW)-fed radiating element with a truncated half-elliptical shape was developed for SWB. In^23^, a circular SWB antenna was developed with a size of 49 mm × 35 mm and its operating range was from 2.5 to 20 GHz. A high bandwidth ratio SWB radiating antenna was developed in^24^, but the MIMO antenna was set up in a 2-D configuration with four ports, resulting in less polarisation flexibility. A spade-shaped SWB diverse antenna along with a decoupling structure in the shape of windmill was developed in^2^. The antenna, measuring 29 mm × 22 mm, operated between 2.9 and 40 GHz frequencies. In^25^, a modified circular monopole antenna was constructed and operates between 2.8 and 40 GHz. Since it was not a MIMO antenna, its signal strength was less due to multipath propagation. In^10^, a bulb-shaped SWB antenna operated between 2.8 and 40 GHz and had an area of 35 mm × 30 mm. In^12^, a single SWB antenna element measured 31 mm × 31 mm and resonated between 2.3 and 18 GHz. According to the literature, even though the presented antennas provide a SWB, they are still difficult to keep compact. However, most of above reported MIMO antennas take up more space and have fewer elements, with polarization vectors restricted to two, and housing effects for SWB antennas are not studied in the literature.

In this article, a thirty-two port SWB diverse antenna in 3-D arrangement is designed. The developed antenna offers a SWB (3 to 40 GHz) with a compact structure, and the arrangement of elements in 3-D form increases the number of polarization vectors. When compared to previous research articles, the obtained antenna gain is significantly high. The housing effect of the antenna is examined by orientating the constructed antenna in an acrylonitrile butadiene styrene (ABS) casing.

## Construction of the thirty-two port diverse antenna

### Unit cell

**Fig. 1:**
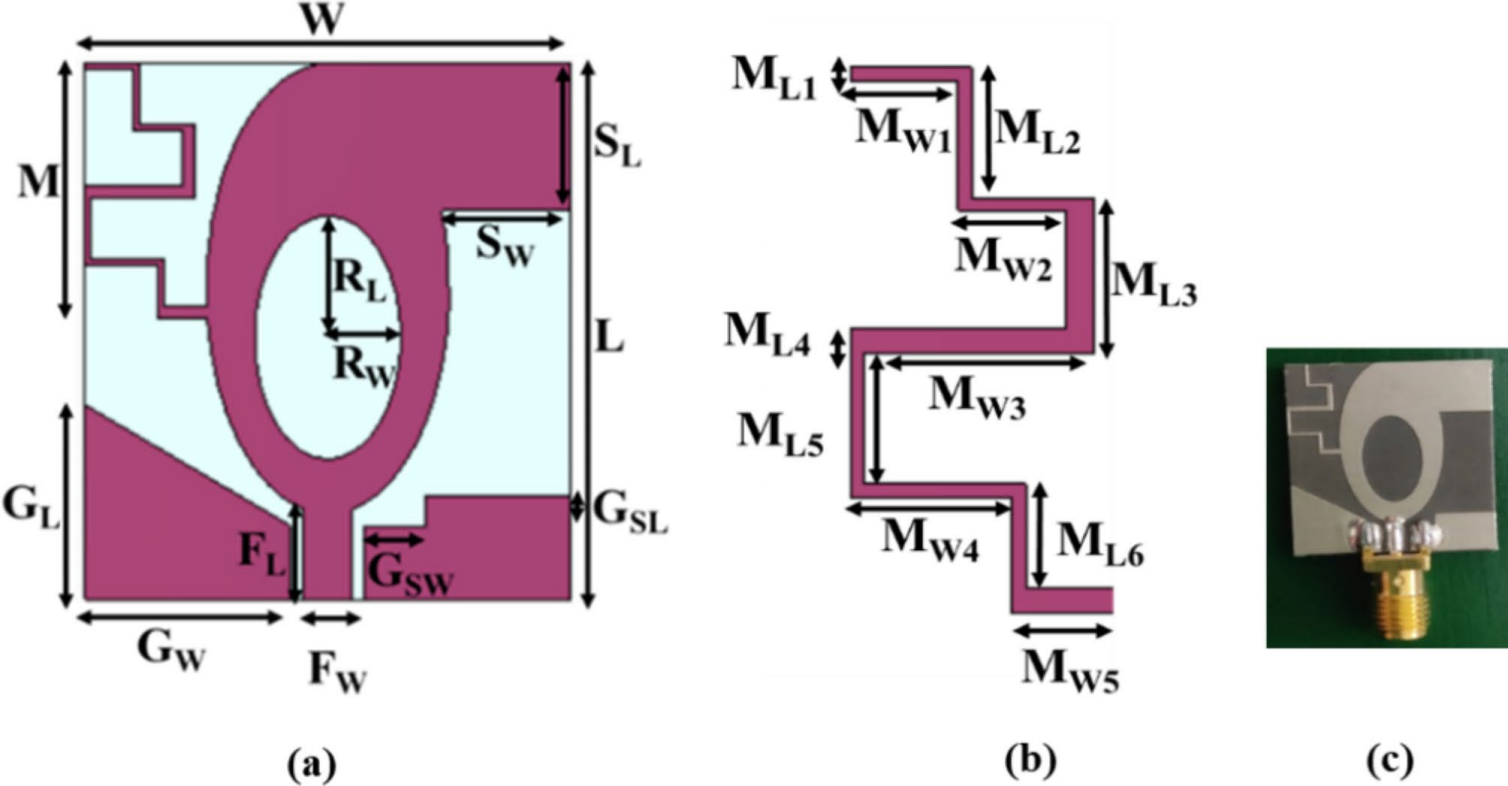
(**a**) Front view of the antenna element, (**b**) Meander line element, (**c**) Fabricated prototype of the antenna element.

The proposed antenna element is constructed on Rogers 5880 substrate as shown in Fig. 1. The substrate has a thickness of 1.52 mm, dielectric constant of 2.2, and loss tangent of 0.0009. The antenna structure is of an elliptical patch connected to a rectangular stub and asymmetric meander line. The antenna is designed using CST Microwave Studio Software. An asymmetric CPW feed supplies power to the unit cell. Figure 1(a) and (b) show the unit cell structure and the asymmetric meander line element. An elliptical slot is introduced within the elliptical patch to increase impedance bandwidth.

**Fig. 2:**
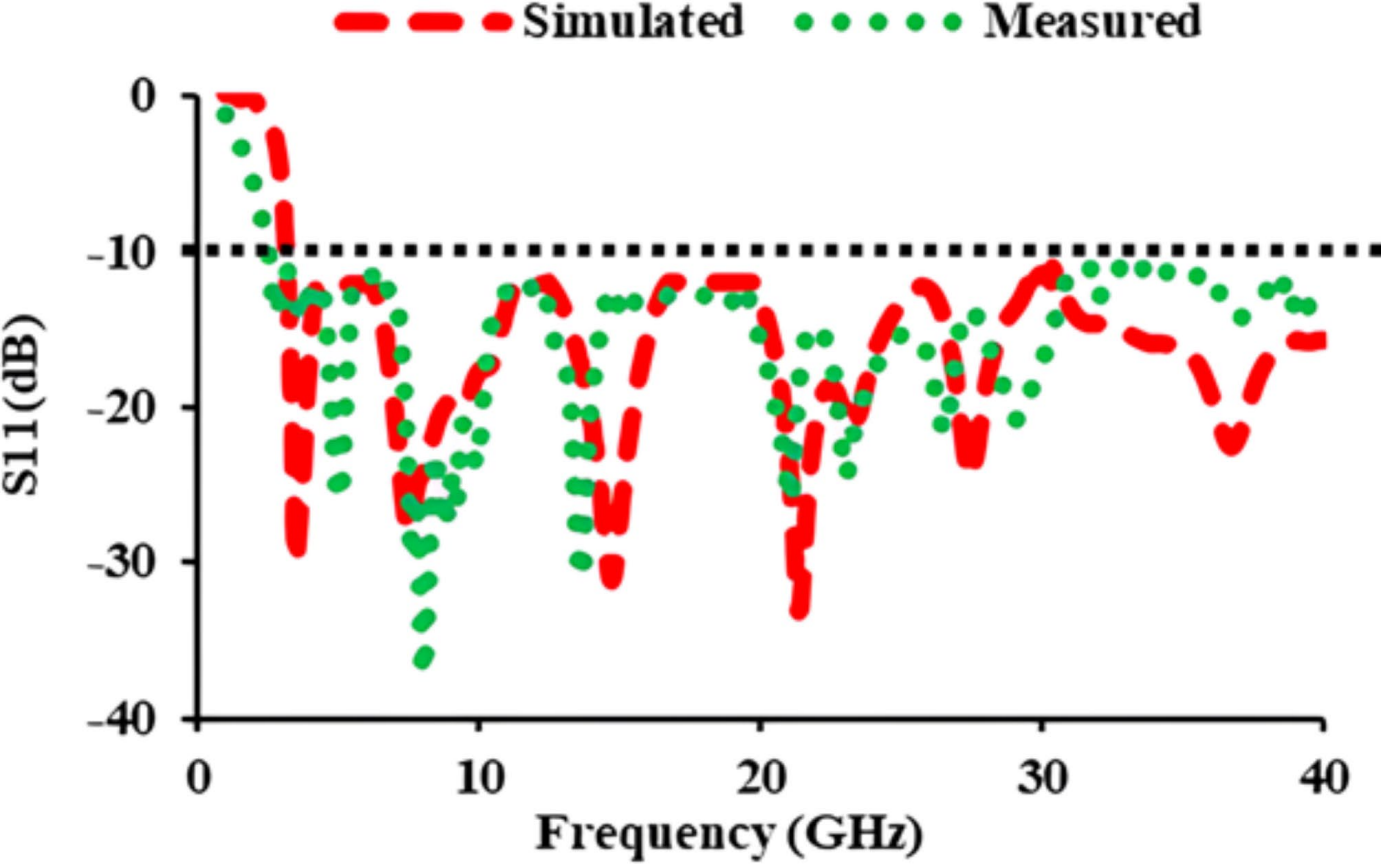
Simulated and measured *S*_11_ values of the antenna element.

In the proposed antenna element, the asymmetric meander line connects the left side of the elliptical patch, while a rectangular stub is included to the right top perspective of the patch. Figure 1(c) demonstrates the manufactured prototype of the antenna element, and it measures 22 mm × 20 mm. Figure 2 displays the simulated and measured *S*_11_-parameters of the antenna element. The plot clearly shows that the *S*_11_ values are lesser than −10 dB from 3 to 40 GHz. Tables 1 and 2 display the structural parameters for the element and asymmetric meander line, respectively.

**Table 1:** Structural parameters of the antenna element.

Notation	Description	Value (mm)
*L*	Length of the element	22
*W*	Width of the element	20
*F*_*L*_	Feed length	3.69
*F*_*W*_	Feed width	2
*R*_*L*_	Radial length of the slot	5.83
*R*_*W*_	Radial width of the slot	3
*M*	Length of the meander line	10.53
*S*_*L*_	Length of the rectangular stub	6
*S*_*W*_	Width of the rectangular stub	5.32
*G*_*L*_	Length of the CPW	8
*G*_*W*_	Width of the CPW	8.5
*G*_*SL*_	Length of the coplanar slot	1.25
*G*_*SW*_	Width of the coplanar slot	2.5

**Table 2:** Design parameter of the asymmetric meander line.

Notation	Value (mm)
*M*_*L*1_	0.25
*M*_*W*1_	2
*ML*_2_	2.5
*MW*_2_	2
*ML*_3_	2.5
*MW*_3_	4.5
*ML*_4_	0.5
*MW*_4_	3
*ML*_5_	2.5
*MW*_5_	2.5
*ML*_6_	2

## Evolution of the developed antenna element

**Fig. 3:**
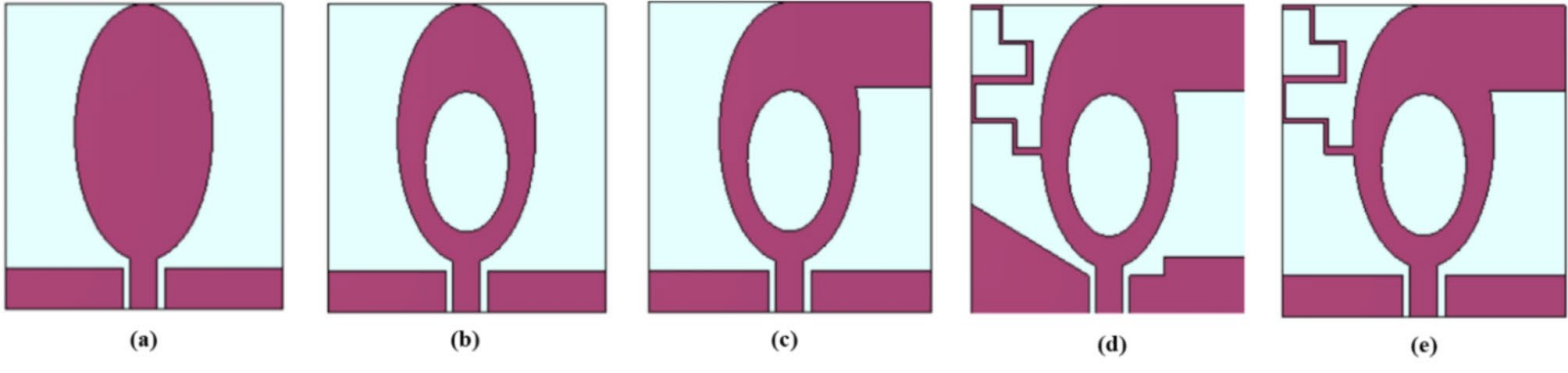
Development stages of the antenna element: (**a**) Stage I, (**b**) Stage II, (**c**) Stage III, (**d**) Stage IV, (**e**) Stage V.

**Fig. 4:**
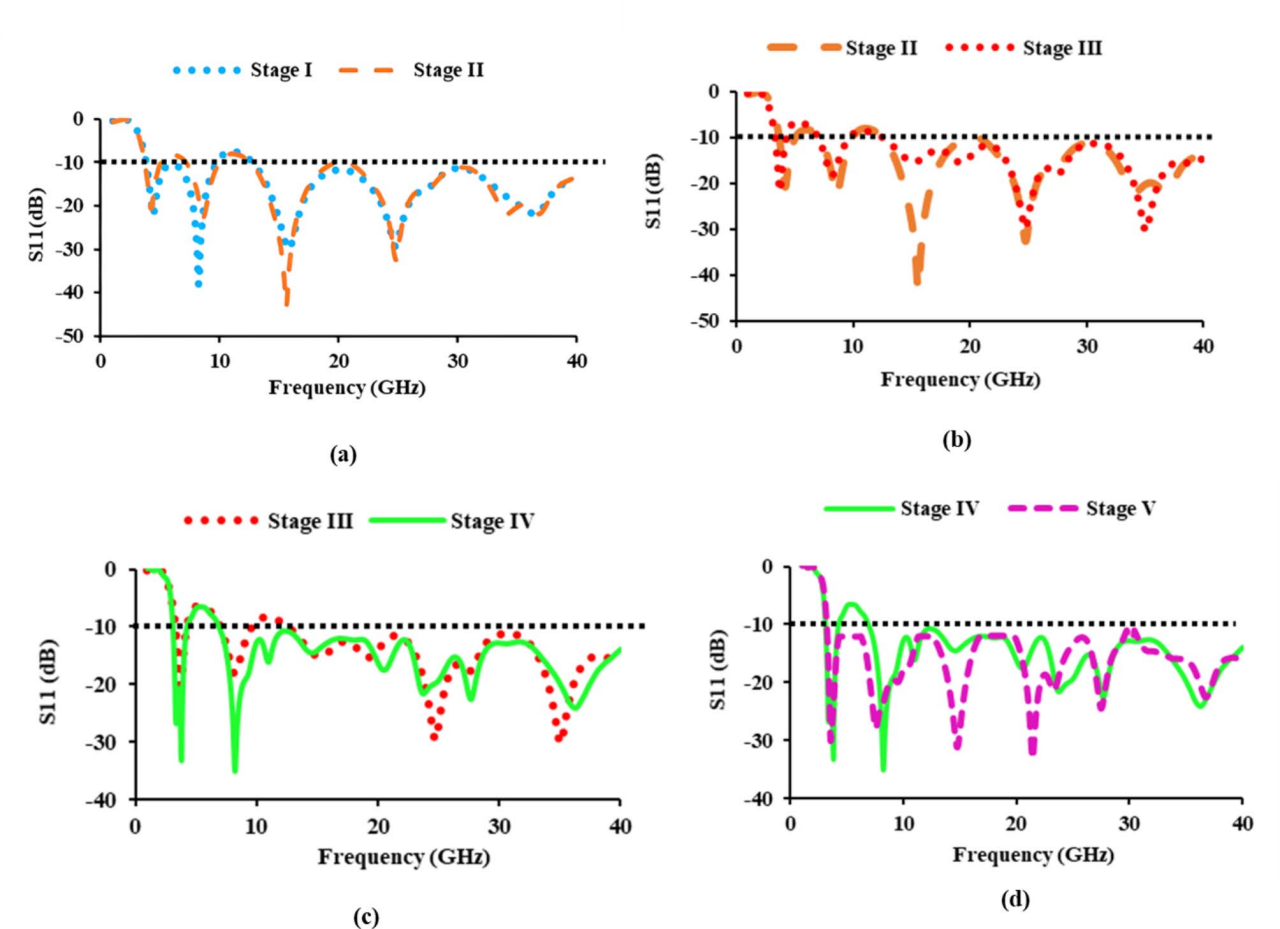
Evolution stages of the antenna element: (**a**) Stage I and stage II, (**b**) Stage II and stage III, (**c**) Stage III and stage IV, (**d**) Stage IV and stage V.

The thirty-two port antenna development stages are demonstrated in Fig. 3, and Fig. 4 shows the respective impedance bandwidth plots. In stage I, an elliptical-shaped patch fed by a CPW is introduced, which offers a bandwidth of 33.14 GHz (3.79 to 9.71 GHz and 12.78 to 40 GHz). When constructing a SWB antenna, it is challenging to achieve a lower frequency range with a compact structure. In stage II, an elliptical slot measuring 5.83 mm × 3 mm is introduced to increase the bandwidth at lower frequencies. The antenna covers frequencies ranging from 3.69 to 5.03 GHz, 7.15 to 9.9 GHz, and 12 to 40 GHz. In stage III, a rectangular stub is added, allowing the antenna to operate between 3.3 and 4.29 GHz, 7 to 9.6 GHz, and 12.63 to 40 GHz. Consequently, when compared to stage II, the 390 MHz frequency shifts to a lower frequency level.

In stage IV, meander lines are introduced to increase the bandwidth in the lower frequency range, resulting in a 330 MHz shift (3 to 4.2 GHz and 6.89 to 40 GHz) in the lower frequency range when compared to stage III. In addition, an impedance bandwidth of 34.31 GHz is achieved, exceeding that of stages I, II, and III. In stage V, an asymmetric CPW is introduced to eliminate notching and achieve a SWB, and the antenna operates from 3 to 40 GHz and higher, covering an impedance bandwidth of 37 GHz and more, which is larger than stages I, II, III, and IV. Thus, the SWB radiating element has evolved to provide a bandwidth ratio greater than 10:1.

An elliptical patch is considered for the proposed SWB antenna as it can provide a wider bandwidth than conventional rectangular or circular patches. It also helps to evenly distribute current across the patch, which improves frequency response. CPW feed is used to feed the patch because it has lower radiation losses than microstrip lines, particularly at higher frequencies. Also, CPW is simple to fabricate as both the patch and ground are in the same plane, and it can be easily integrated with other electronic circuits.

An elliptical slot is introduced as it affects current distribution and provides a wider bandwidth. A rectangular stub is introduced because it has frequency tuning capabilities. By varying the dimensions of the stub, the resonant frequency can be shifted to achieve the desired operating frequency. The position and length of the rectangular stub play a major role in impedance matching. The introduction of meander lines helps in tuning the antenna to a lower frequency as the electrical length increases. An asymmetric CPW is used to provide a wide bandwidth. The asymmetry structure alters the current distribution, increasing the operational frequency range of the antenna.

## Surface current distribution

**Fig. 5:**
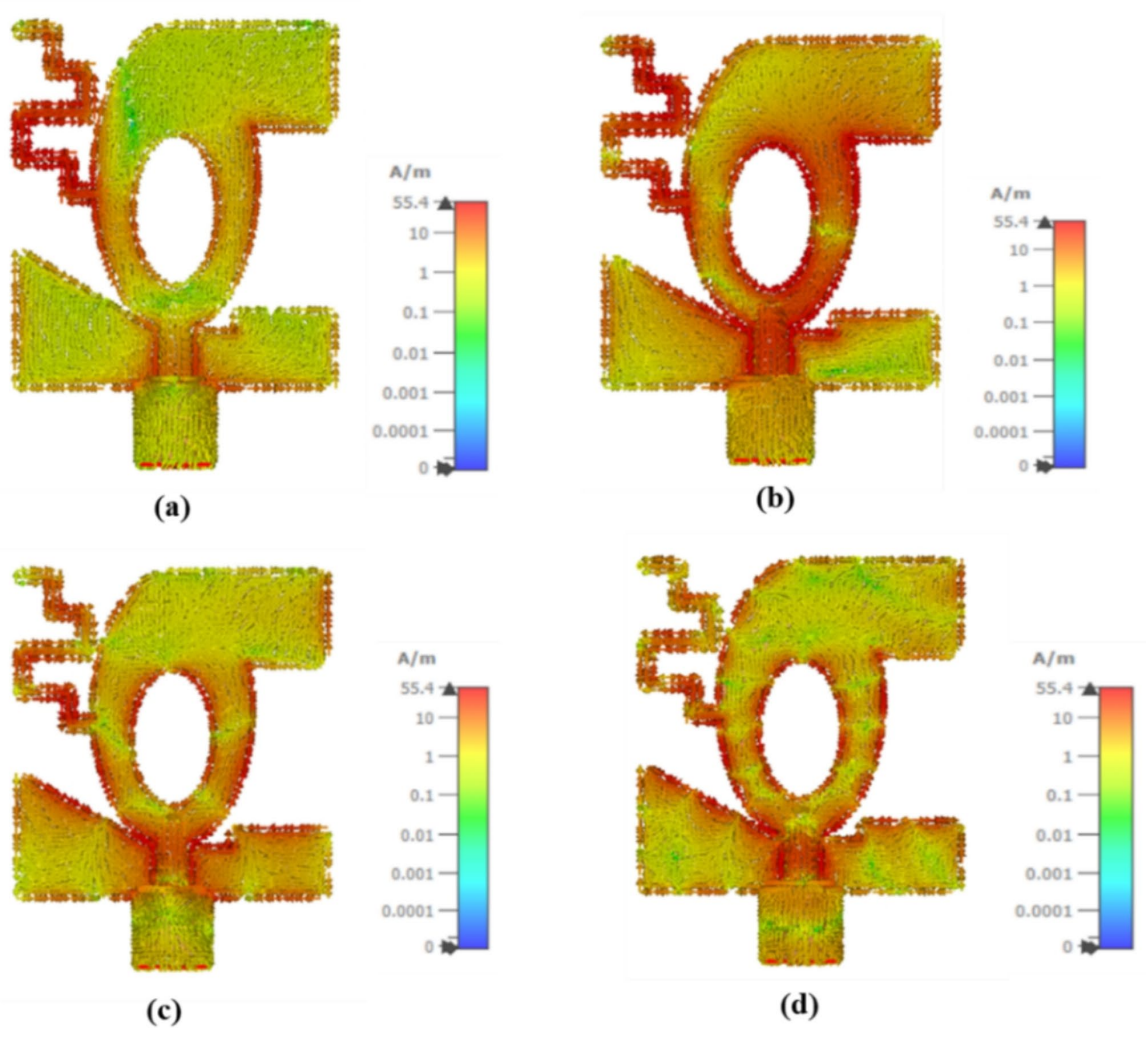
Surface current density of the developed antenna element: (**a**) 3.8 GHz, (**b**) 10 GHz, (**c**) 20 GHz, (**d**) 40 GHz.

The surface current distribution of the developed antenna element is demonstrated in Fig. 5. The surface current distribution is observed at 3.8 GHz, 10 GHz, 20 GHz, and 40 GHz. Figure 5 shows that at 3.8 GHz, the current density is concentrated in the lower region of the asymmetric meander lines, while at 10 GHz, the current density is denser on the right perspective of the patch and meander lines. At 20 GHz, the current density is large in the coplanar region, while at 40 GHz, the current density is denser in the upper region of the patch.

## MIMO antenna

**Fig. 6:**
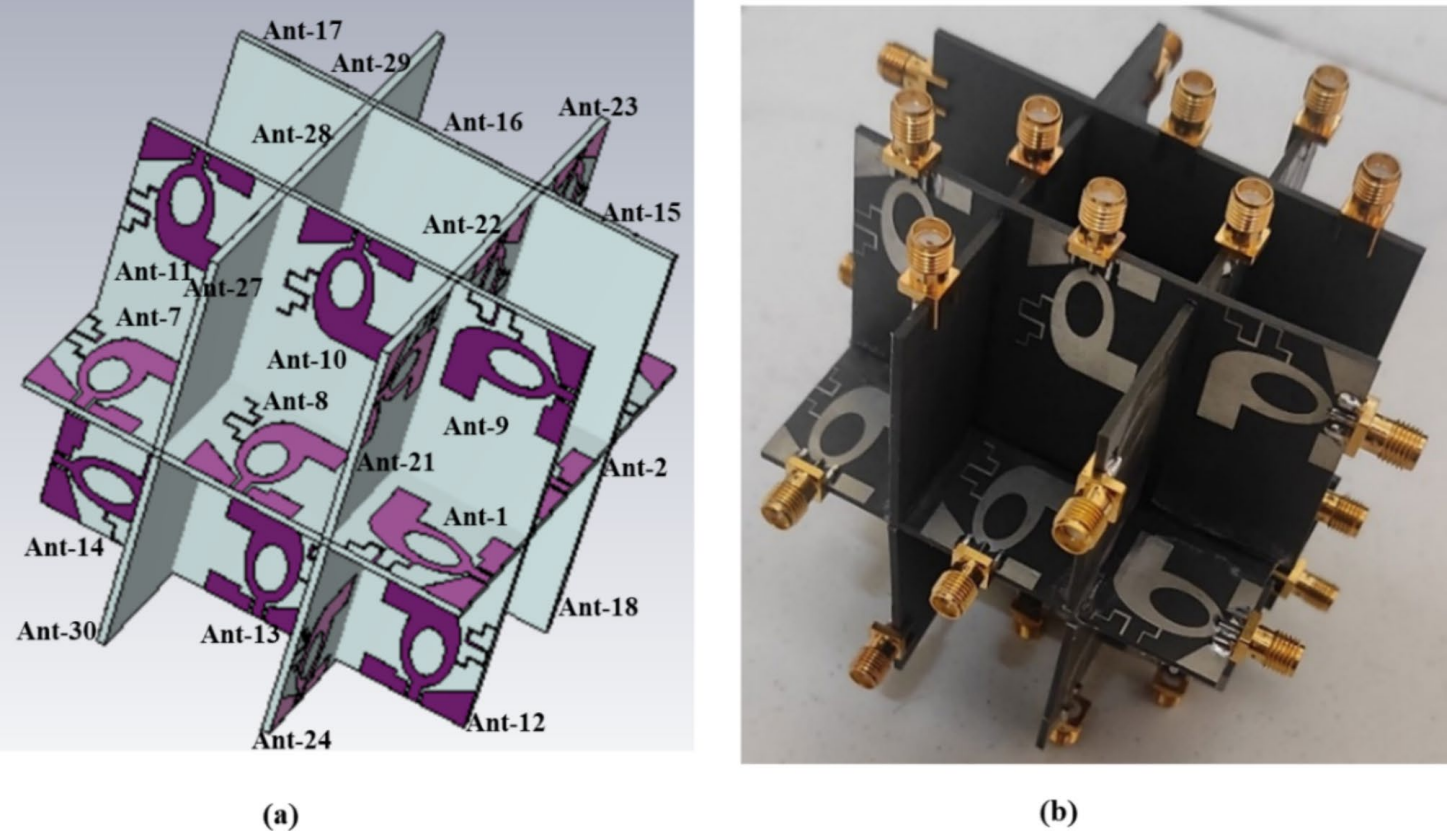
(**a**) Design of thirty-two port diverse antenna, (**b**) Fabricated prototype.

The designed antenna element is developed into a thirty-two port diverse antenna, as shown in Fig. 6(a). When viewed from the front, the constructed MIMO antenna is made up of five planes: one is the base plane, two are in the direction of X, and two are in the Y-direction. The base plane consists of eight elements, the third of which is orthogonal to the first and second. Element 5 is orthogonal to elements 3 and 4, element 7 is orthogonal to elements 5 and 6, and element 1 is orthogonal to elements 7 and 8.

The four planes in the X- and Y-directions each contain six elements. The X and Y planes are perpendicular to the base plane, with three elements above and three elements below. There are two X planes and two Y planes, identified as X_plane_1, X_plane_2, Y_plane_1, and Y_plane_2. In X_plane_1, element 9 is orthogonal to elements 10 and 11, and element 12 to elements 13 and 14. In the second X plane, element 15 is orthogonal to elements 16 and 17, and element 18 is orthogonal to elements 19 and 20. In Y_plane_1, element 21 is orthogonal to elements 22 and 23, while element 24 is orthogonal to elements 25 and 26. In the second Y plane, element 27 is orthogonal to elements 28 and 29, and element 30 is orthogonal to elements 31 and 32.

The isolation between the elements must be increased for better diverse antenna performance, while the envelope coefficient correlation (ECC) has to be reduced. To achieve good isolation, the proposed thirty-two port diverse antenna maintains 10 mm distance between each element. Figure 6(b) demonstrates the manufactured prototype of the thirty-two port diverse antenna, and it measures 82 mm × 82 mm.

## Results and inferences

### Radiation properties of the antenna

The developed thirty-two port SWB diverse antenna is manufactured and examined, and its simulated and measured characteristics are discussed here.

### *S*-parameters of the antenna

**Fig. 7:**
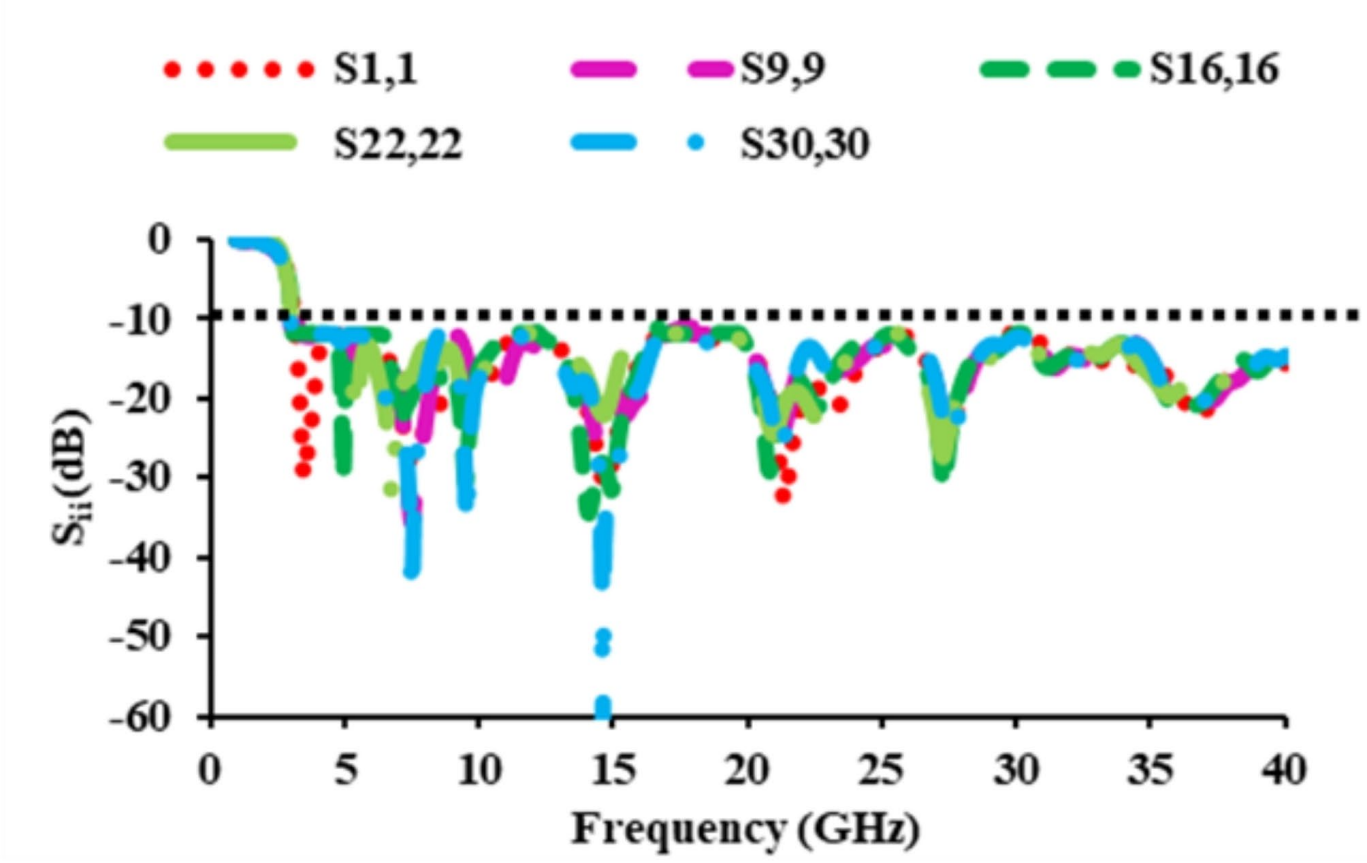
*S*_*ii*_ parameters of the thirty-two port diverse antenna.

The simulated as well as measured reflection coefficients of the constructed single element antenna are displayed in Fig. 2. The reflection coefficients are found to be less than −10 dB from 3 to 40 GHz. Figure 7 depicts the *S*_*ii*_ characteristics of the thirty-two port MIMO antenna. The *S*_*ii*_ of the designed antenna are plotted in five different planes. The *S*_*ii*_ characteristics of antenna_1 of the base plane, antenna_9 of the X_plane_1, antenna_16 of the X_plane_2, antenna_22 of the Y_plane_1, and antenna_30 of the Y_plane_2 are plotted. Figure 7 displays that the proposed thirty-two port SWB MIMO resonates from 3 to 40 GHz.

**Fig. 8:**
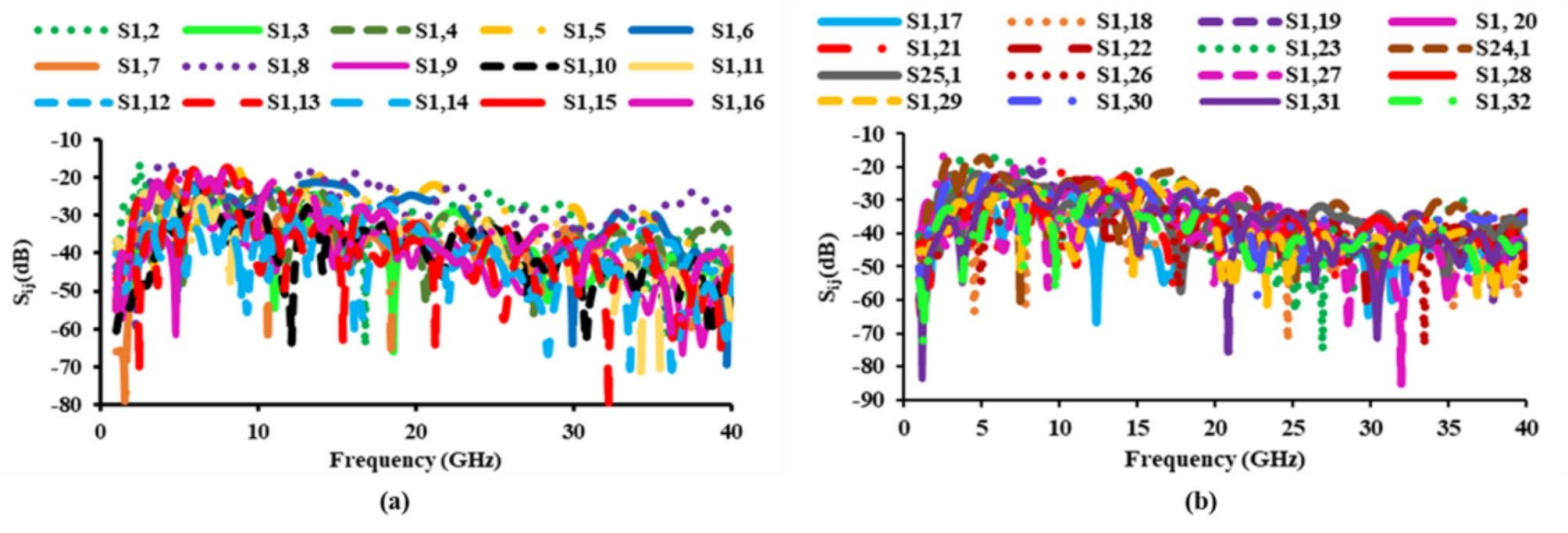
Mutual coupling of the proposed diverse antenna: (**a**) *S*_*ij*_ of port 1 to 16, (**b**) *S*_*ij*_ of port 17 to 32.

**Fig. 9:**
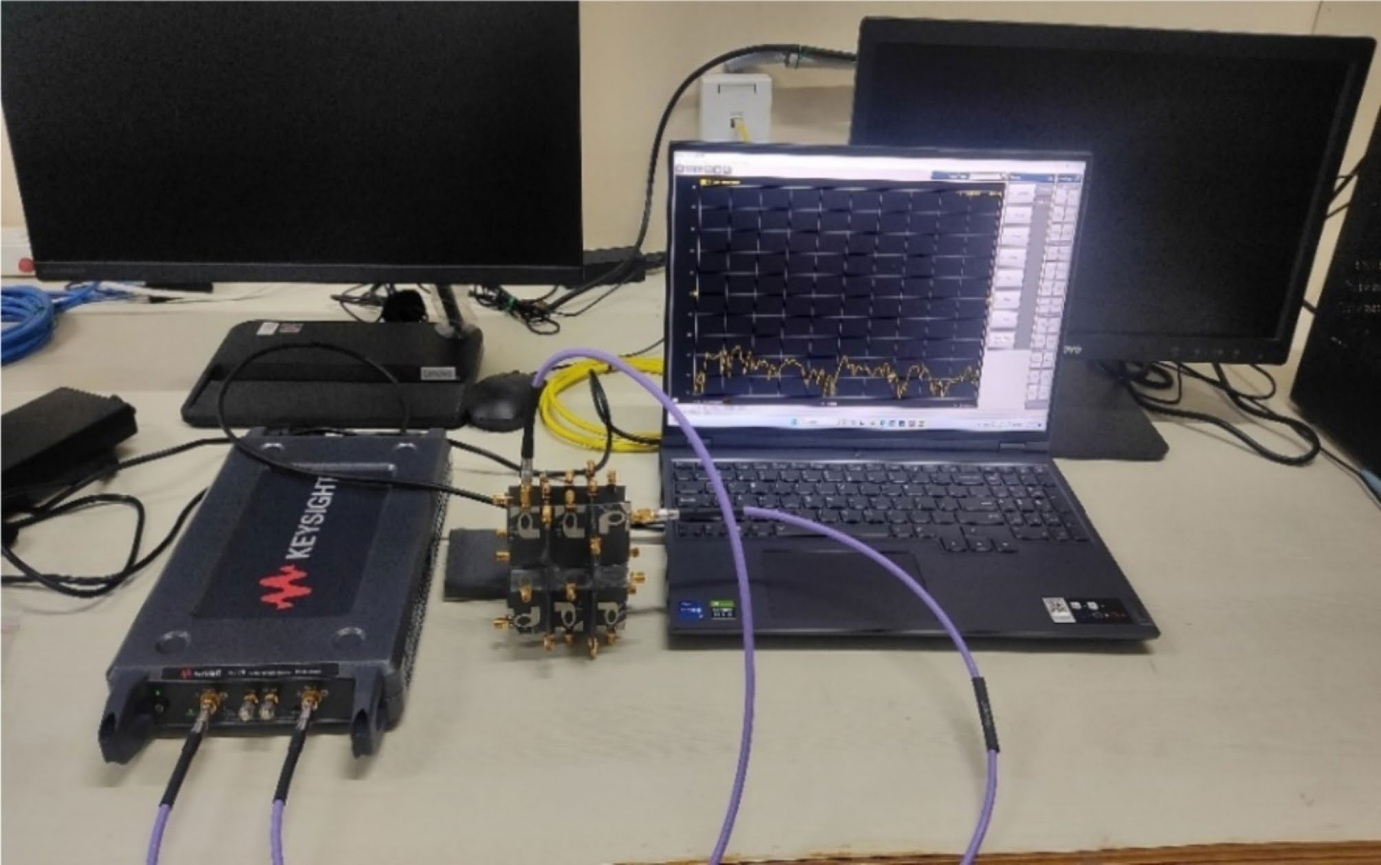
Testing of the proposed MIMO antenna.

Another factor to account for in MIMO antenna design is mutual coupling.The *S*_*ij*_ characteristics of the developed antenna for antenna_1 are demonstarted in Fig. 8. The mutual coupling value is found to be lower than −15 dB at all ports. The *S*-parameters of the constructed antenna are tested using a vector network analyzer and the graphs are visualized using a laptop, as illustrated in Fig. 9.

## Bandwidth ratio, fractional bandwidth (FBW), and bandwidth dimensional ratio (BDR)

Bandwidth ratio is defined as the ratio of lower to higher cut-off frequencies. A SWB antenna should have a bandwidth ratio of at least 10:1. Fractional bandwidth (FBW) refers to the difference between the higher and lower cut-off frequencies divided by the centre frequency. The expression of the FBW is given as,

1 $$FBW=\frac{{f}_{2}-{f}_{1}}{{f}_{c}}$$

where upper cut-off frequency is represented as *f*_2_, *f*_1_ is the minimal cut-off frequency, and *f*_*c*_ is the mid frequency. The fractional bandwidth typically ranges from 0 to 2 and is commonly represented as a percentage between 0% and 200%. If the FBW is greater than 20%, it indicates a wideband, and for UWB, it must be greater than 50%.

The bandwidth dimensional ratio (BDR) is used to verify size, efficiency, and bandwidth of the antenna. It calculates the amount of fractional operational bandwidth allocated per electrical unit area. The expression of the BDR is given as^26^,

2 $$BDR=\frac{BW\%}{{\lambda}_{L}\times{\lambda}_{W}}$$

where ${\lambda}_{L}$is the length of the antenna element (in terms of wavelength), ${\lambda}_{W}$ is the width of the antenna element (in terms of wavelength), and *BW* is the bandwidth. The BDR for the designed unit cell of the thirty-two port SWB antenna is 3911. If the BDR value is high, it will cover a wide range while remaining compact in size. The proposed unit cell has been simulated up to 100 GHz, and the impedance bandwidth is found to be lower than −10 dB from 3 to 100 GHz, resulting in BDR and FBW greater than 33:33:1 and 189.45%, respectively. However, the developed antenna is limited to 40 GHz because of the computational time and measurement facilities constraints. The obtained BDR and FBW of the developed MIMO antenna are 13:33:1 and 172.09%, respectively.

### Radiation patterns

**Fig. 10:**
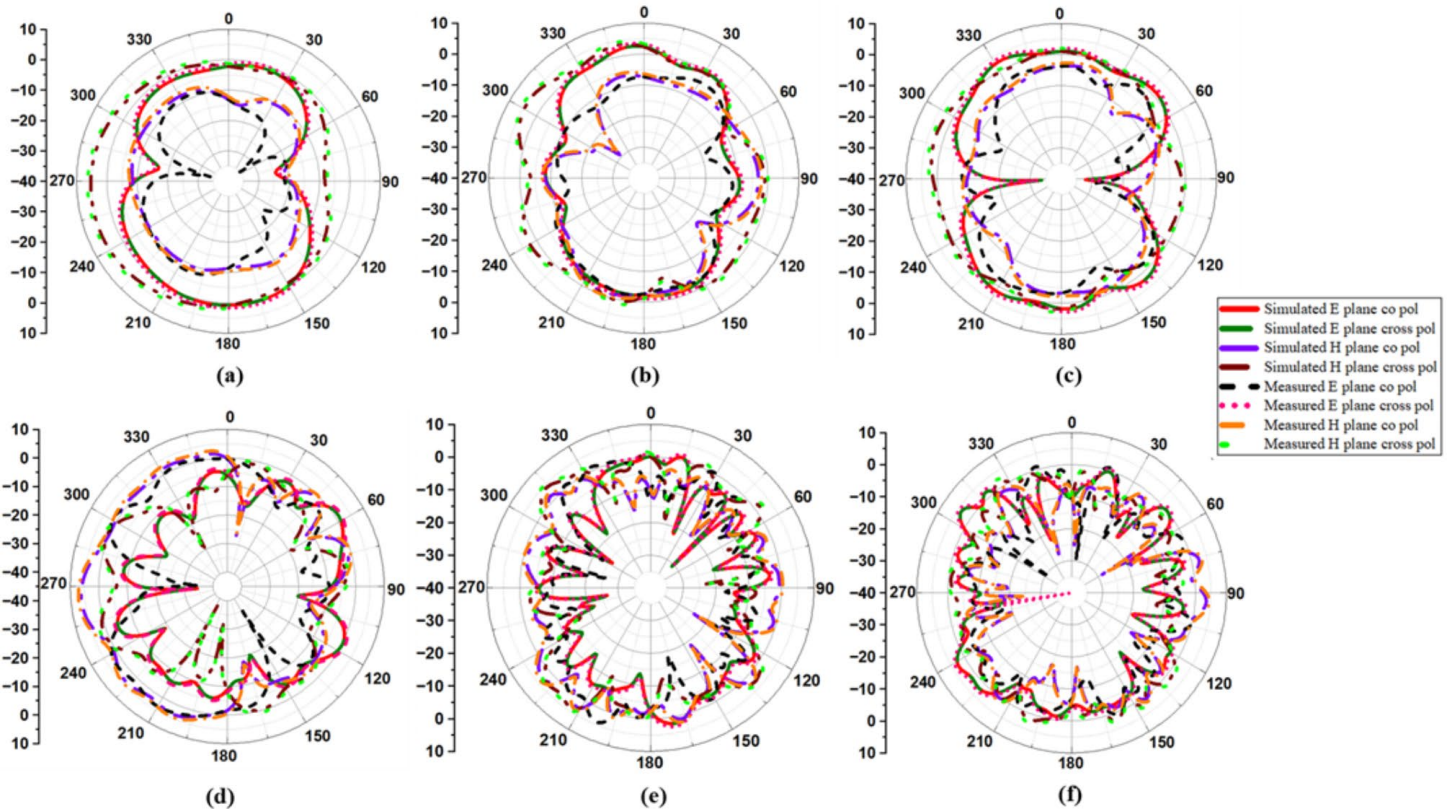
Co- and cross-polarization of antenna_1: (**a**) 3.8 GHz, (**b**) 5.4 GHz, (**c**) 6.1 GHz, (**d**) 10.1 GHz, (**e**) 17.5 GHz, (**f**) 20.5 GHz.

**Fig. 11:**
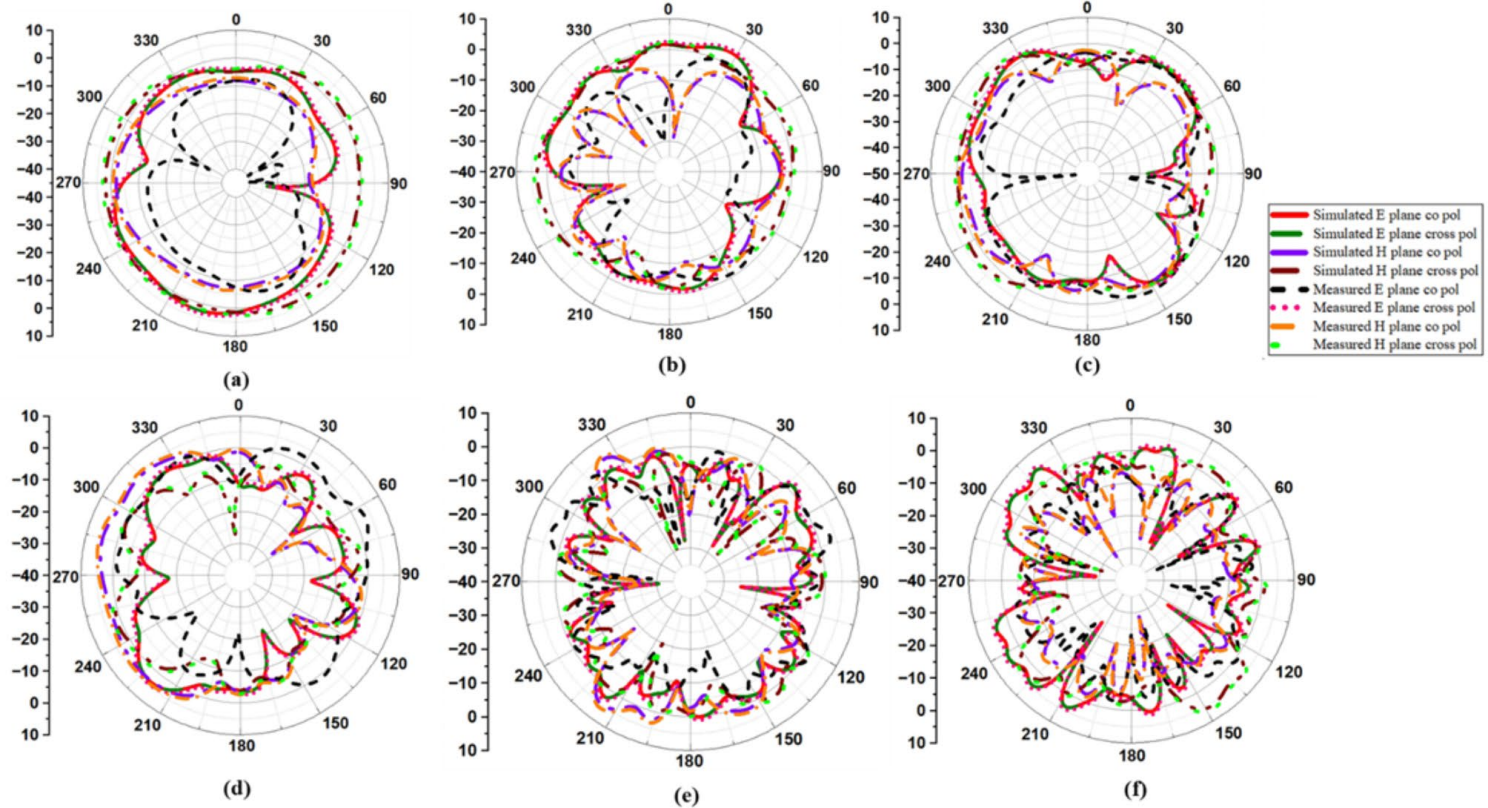
Co- and cross-polarization of antenna_2: (**a**) 3.8 GHz, (**b**) 5.4 GHz, (**c**) 6.1 GHz, (**d**) 10.1 GHz, (**e**) 17.5 GHz, (**f**) 20.5 GHz.

**Fig. 12:**
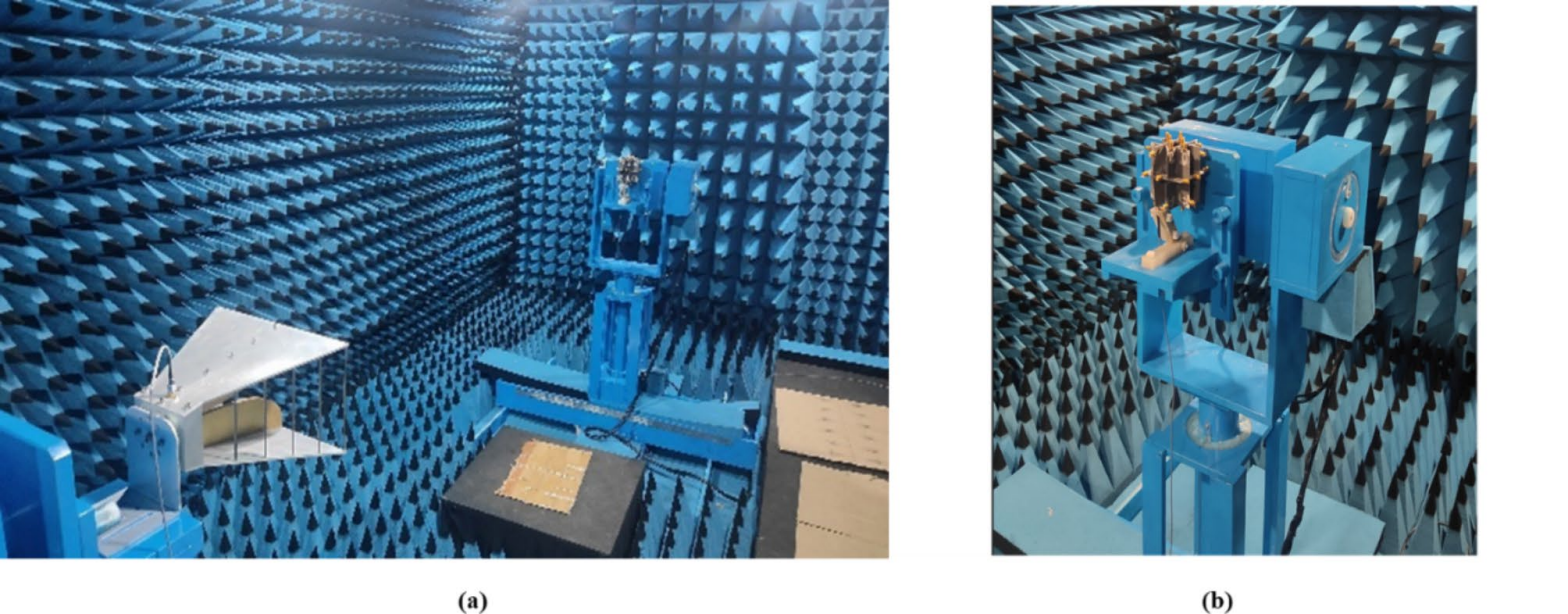
Testing of thirty-two port antenna in an anechoic chamber: (**a**) Transmitting horn antenna, (**b**) Receiving antenna.

Figure 10 depicts the simulated and measured co- and cross-polarization of suggested antenna_1 in the E- and H-planes at spectral bands of 3.8 GHz, 5.4 GHz, 6.1 GHz, 10.1 GHz, 17.5 GHz, and 20.5 GHz, while Fig. 11 depicts the radiation distribution of antenna_2. The co- and cross-polarization of the designed antenna are tested in an anechoic chamber, as shown in Fig. 12(a) and (b). The developed antenna is used as the receiver antenna, while the traditional horn antenna serves as the transmitter. Each element of the antenna radiates a unique radiation distribution, and the loss of gain of one element is compensated for by the other elements.

**Fig. 13:**
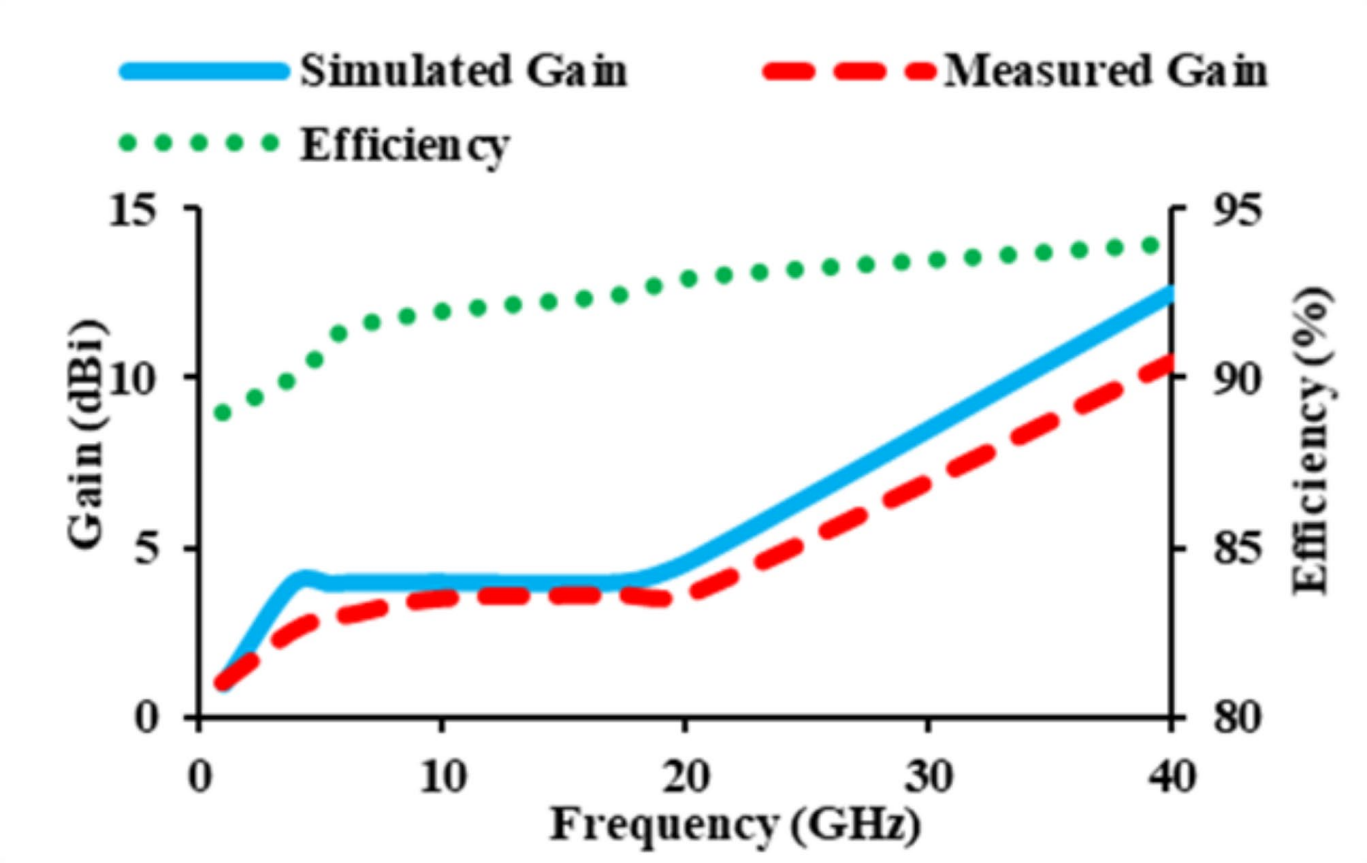
Gain and efficiency of the thirty-two port diverse antenna.

The gain characteristics and efficiency of the antenna are displayed in Fig. 13. The constructed antenna has a maximum gain of 12.5 dBi at 40 GHz. The gain obtained is above 3.9 dBi across the entire operating frequency range (3 to 40 GHz). The maximum efficiency of the constructed antenna is 94%.

### MIMO characteristics

This section examines the diverse properties of the suggested antenna, including ECC, total active reflection coefficient (TARC), diversity gain (DG), mean effective gain (MEG), and channel capacity loss (CCL).

**Fig. 14:**
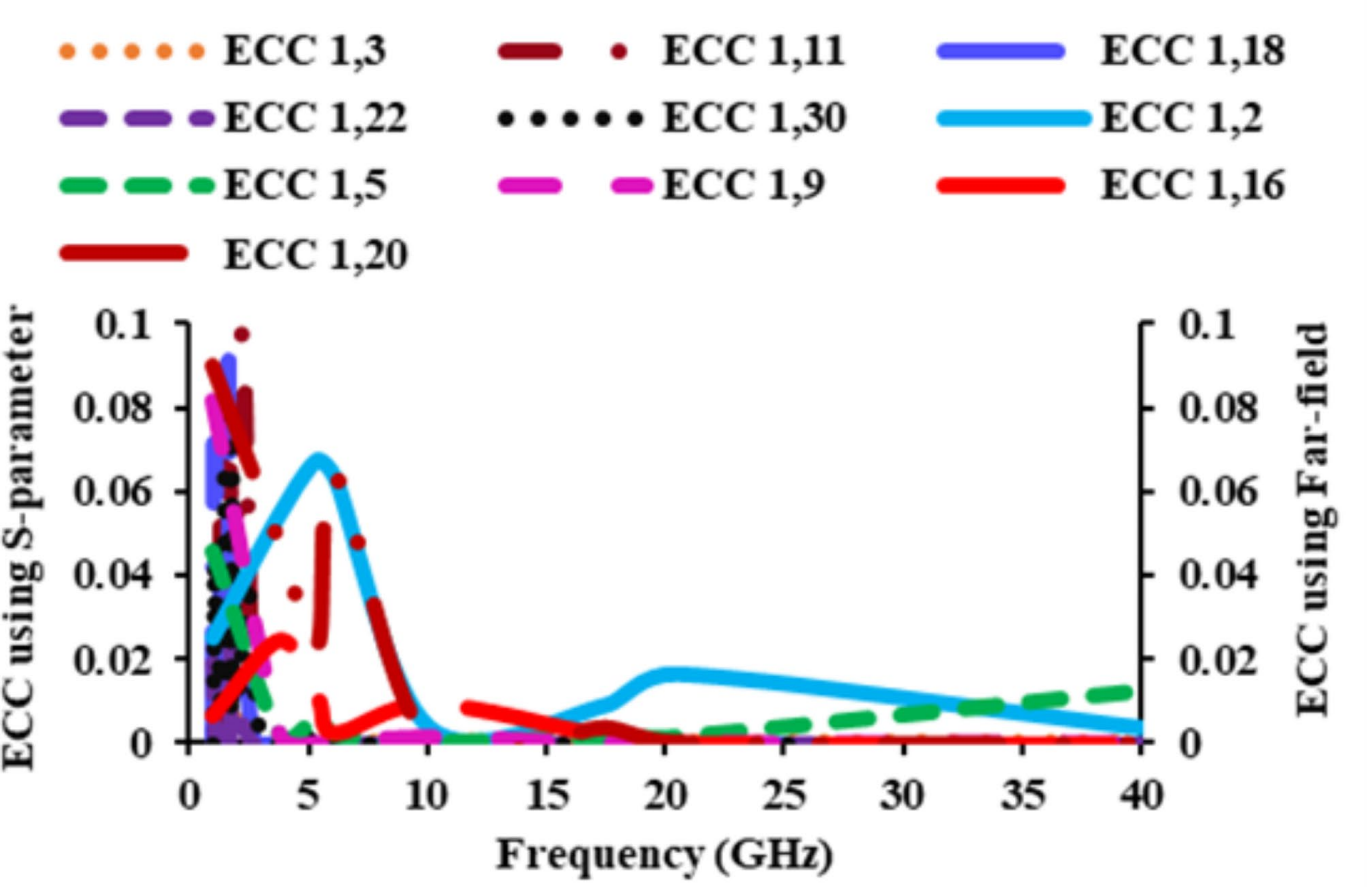
ECC of the suggested antenna (*S*-parameter and far-field).

ECC can be defined as the degree of isolation amidst dual elements of a diverse antenna. The antenna’s ECC must be zero ideally, but in real time scenarios, it should be minimal than 0.5. The ECC calculation involves using the *S*-parameter and the far-field as^17^,

3 $$ECC\left({\rho}_{e}\right)=\frac{{|{S}_{ii}^{*}{S}_{ij}+{S}_{ji}^{*}{S}_{jj}|}^{2}}{\left(1-{\left|{S}_{ii}\right|}^{2}-{\left|{S}_{ij}\right|}^{2}\right)\left(1-{{|S}_{ji}|}^{2}-{{|S}_{ii}|}^{2}\right)}$$

4 $$ECC{(\rho}_{e})=\frac{{\left|\iint\left[{\overrightarrow{F}}_{1}\left(\theta,\left.\phi\right).{\overrightarrow{F}}_{2}\left(\theta,\phi\right)]d\varOmega\right.\right.\right.|}^{2}}{\iint{\left|{\overrightarrow{F}}_{1}\left(\theta,\phi\right)\right|}^{2}d\varOmega\iint{\left|\overrightarrow{{F}_{2}}\left(\theta,\phi\right)\right|}^{2}d\varOmega}$$

where *S*_*ii*_ represents the *S*-parameters of the antenna, *S*_*ij*_ indicates the correlation of *S*-parameters of two different antenna elements, and these elements radiated field is represented by *F*, *θ* represents elevation angle, azimuth angle is given by *φ*, and solid angle is denoted by *Ω*. Figure 14 shows that the obtained ECC values are well below 0.1, which is the practical limit, and thus the antenna’s performance will not be affected.

The DG reduces transmission power while maintaining performance and increases the signal-to-interference ratio. A diverse antenna’s DG value ought to be higher than 8.86 dB in practical cases. Both the far-field and the *S*-parameter can be used to determine the DG. The DG can be expressed as,

5 $$DG=10\sqrt{1-{\left|{\rho}_{e}\right|}^{2}}$$

**Fig. 15:**
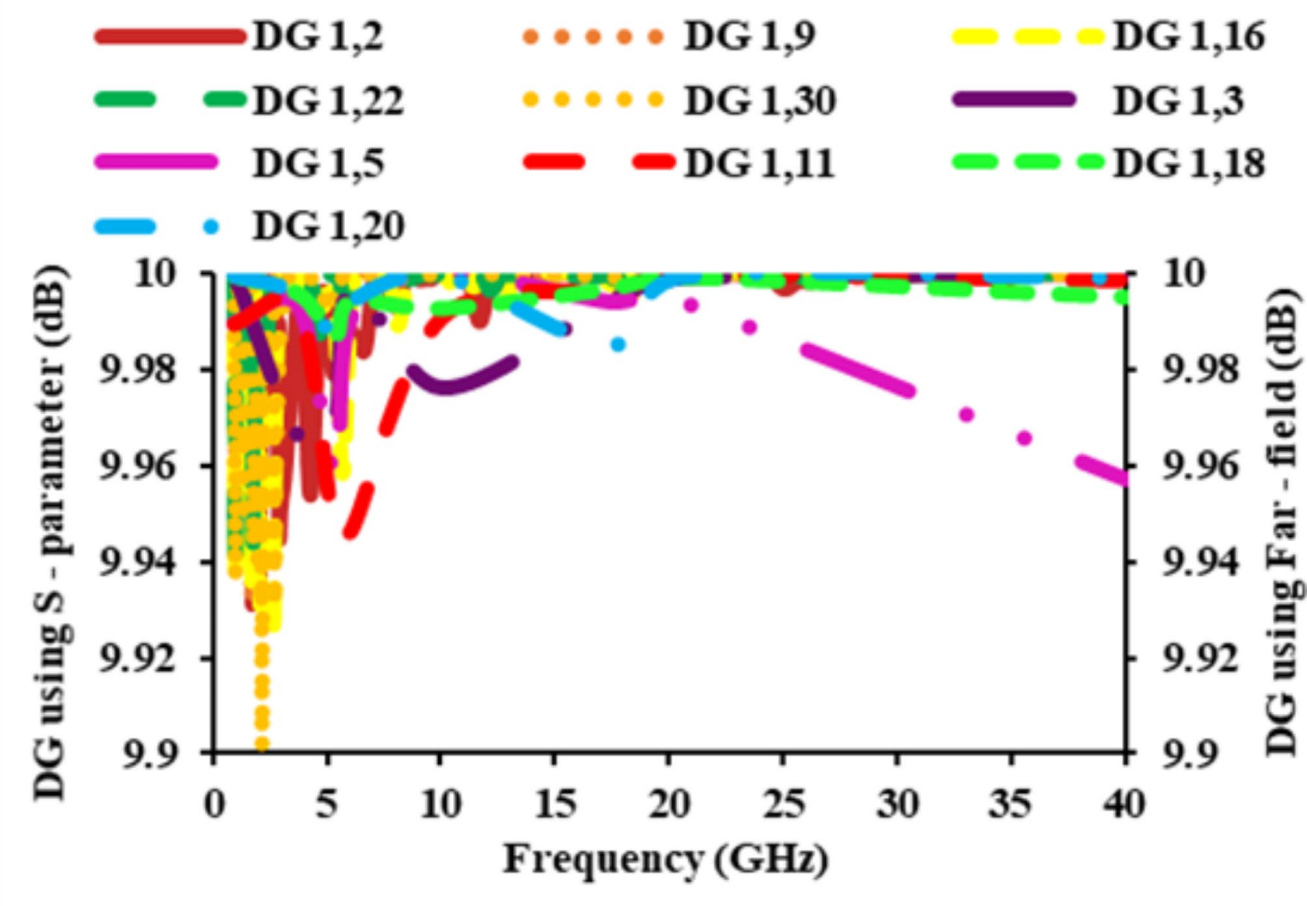
DG of the diverse antenna.

The obtained DG of the proposed MIMO antenna is plotted in Fig. 15. In the majority of cases, the DG is equal to 10 dB, and the attained DG values exceed 9.9 dB. The graph clearly shows that the attained DG is within the desired limit.

MEG is defined as as the capability of the antenna to acquire transmitted electromagnetic waves. It is computed in far-field, and its practical limit is less than 3 dB. MEG can be expressed mathematically using Eq. (6).

6 $$MEG=\int_{0}^{2\pi}\int_{0}^{\pi}{\left[\frac{XPR}{1+XPR}{G}_{\theta}\left(\theta,\varPhi\right){P}_{\theta}\left(\theta,\varPhi\right)+\frac{1}{1+XPR}{G}_{\varPhi}\left(\theta,\varPhi\right){P}_{\varPhi}\left(\theta,\varPhi\right)\right]{\sin}\, \theta d\theta d\varPhi}$$

Table 3 shows the MEG for the developed antenna with respect to port-1. Table 3 shows that the MEG is below 3 dB, meeting the practical limits. The results are validated for isotropic, outdoor, and indoor scenarios with XPR values of 0, 1, and 5, respectively.

**Table 3:** MEG of the suggested antenna in relation to port-1.

Frequency (GHz)	XPR	MEG 1/2	MEG 1/16	MEG 1/30
**10.1**	0 (isotropic)	0.89	0.63	0.79
	1 (outdoor)	0.90	0.70	0.81
	5 (indoor)	0.93	0.98	0.9
**20.5**	0 (isotropic)	0.88	1.05	1.16
	1 (outdoor)	0.91	1.13	1.28
	5 (indoor)	1.01	1.47	1.83
**40**	0 (isotropic)	0.92	0.84	0.73
	1 (outdoor)	0.93	0.82	0.74
	5 (indoor)	0.93	0.75	0.73

**Fig. 16:**
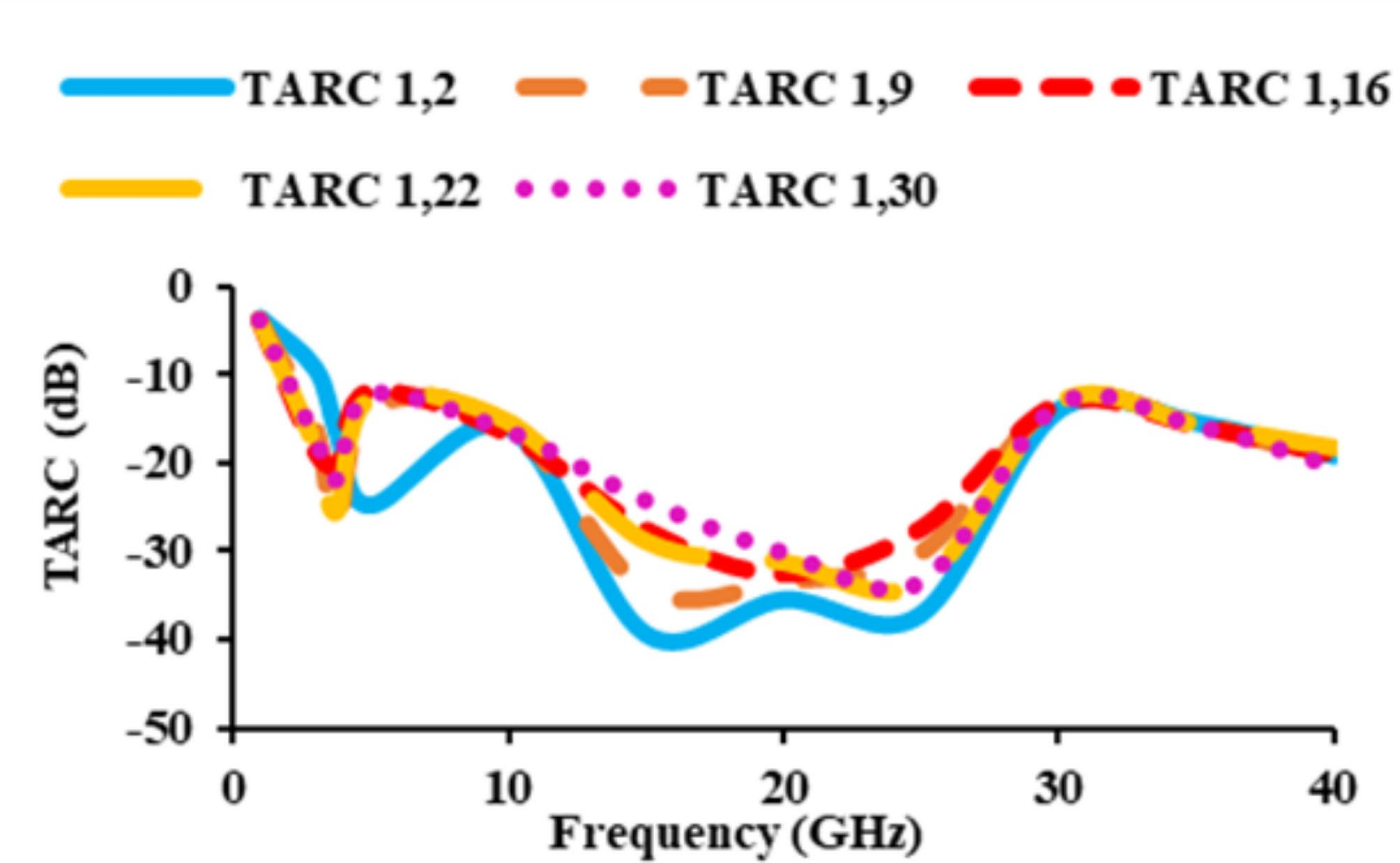
TARC of the suggested thirty-two port SWB antenna.

The TARC of a MIMO antenna is described as the ratio of the square root of reflected power to the square root of incident power. The incident signal is denoted by *a*_*i*_, while the reflected signal is represented by *b*_*i*_. The TARC must be minimal than −10 dB. The smaller the TARC, the lower the mutual coupling, and higher the isolation. Therefore, the obtained TARC value should be low, and thus the isolation increases. Figure 16 shows that the obtained TARC is minimal than −10 dB.

7 $$TARC=\frac{\sqrt{{\sum}_{i=1}^{N}{\left|{b}_{i}\right|}^{2}}}{\sqrt{{\sum}_{i=1}^{N}{\left|{a}_{i}\right|}^{2}}}$$

The capacity loss caused by correlation in MIMO channels is investigated using CCL, which is calculated as,

8 $$CCL=-{{\log}}_{2}\left|{\psi}^{R}\right|$$

9 $${\psi}^{R}=\left[\begin{array}{cc}{\rho}_{ii}&{\rho}_{ij}\\{\rho}_{ji}&{\rho}_{jj}\end{array}\right]$$

where $${\rho}_{ii}=\left(1-{\left|{S}_{ii}\right|}^{2}-{\left|{S}_{ij}\right|}^{2}\right),{\rho}_{22}=\left(1-{\left|{S}_{jj}\right|}^{2}-{\left|{S}_{ji}\right|}^{2}\right),$$ and$${\rho}_{ij}=-\left({S}_{ii}^{*}{S}_{ij}+{S}_{ji}^{*}{S}_{ij}\right)\; {\text{and}}\; {\rho}_{ji}=-\left({S}_{jj}^{*}{S}_{ji}+{S}_{ij}^{*}{S}_{ji}\right).$$

**Fig. 17:**
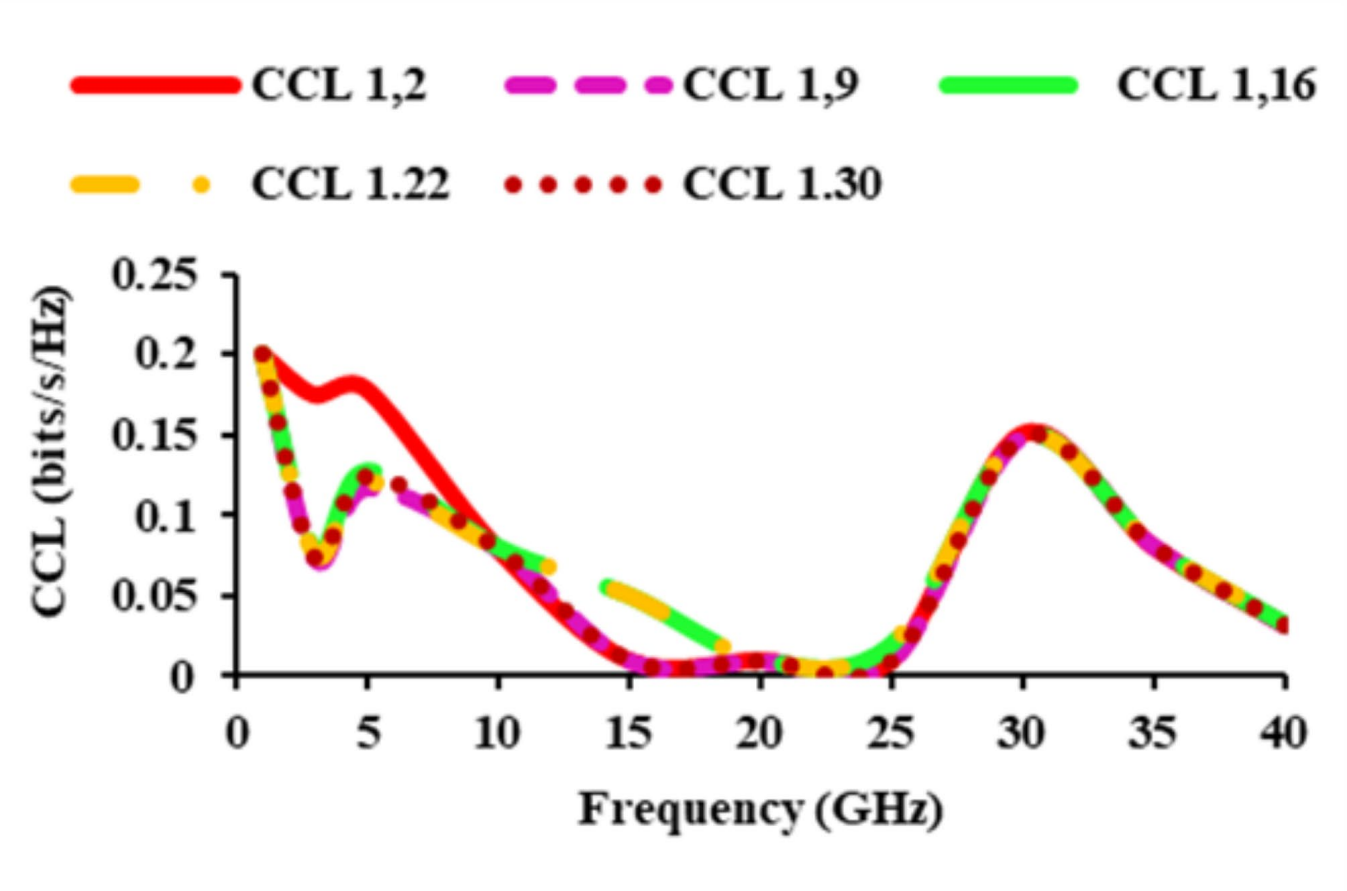
CCL of the suggested thirty-two port SWB antenna.

CCL of the diverse antenna should be lower than 0.4 bits/s/Hz. Figure 17 shows that the CCL of the designed antenna is below 0.2 bits/s/Hz.

**Fig. 18:**
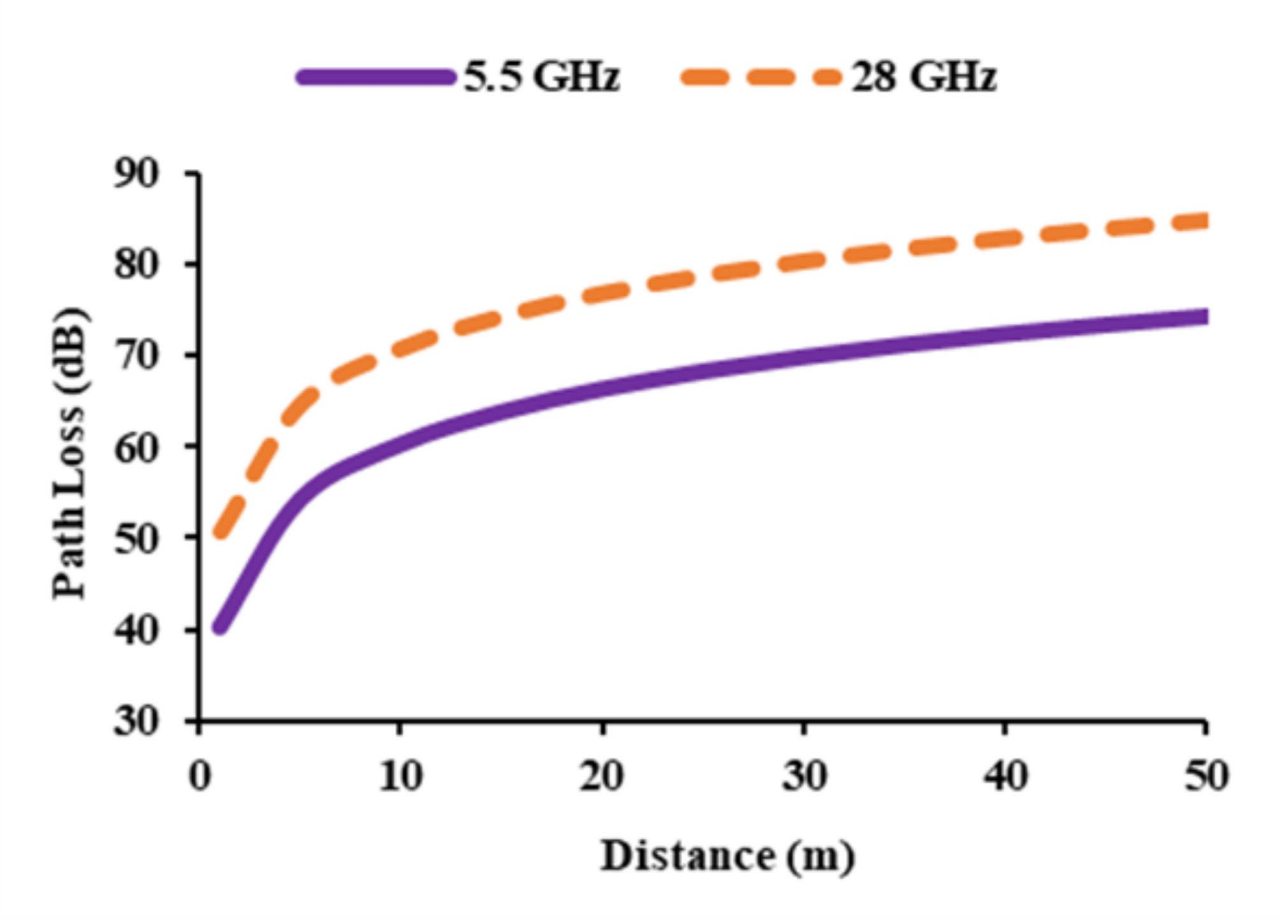
Path loss of the thirty-two port diverse antenna at 5.5 GHz and 28 GHz.

Path loss analysis examines the signal propagation or attenuation that occur between the transmitting and receiving antennas, taking into account the received signal strength and the corresponding path loss. The path loss is computed for the constructed antenna resonating at of 5.5 GHz and 28 GHz. The separation between the transmitting and receiving antennas is varied up to 50 m, and the resulting path loss is computed. The transmitting gain of the antenna is 3 dBi at both frequencies^26,27^. The gain of the receiver antenna is 3.95 dBi at 5.5 GHz and 7.5 dBi at 28 GHz, respectively. Equation (10) is used to calculate free space path loss (FSPL).

10 $$FSPL=20 {log}_{10}\left(d\right)+20 {log}_{10}\left(f\right)+20 {log}_{10}\left(\frac{4\pi}{c}\right)-{G}_{Tx}-{G}_{Rx}$$

where *d* is the separation between the transmitting and receiving antennas, frequency is denoted as *f*, *G*_*Tx*_ is the gain of the transmitting antenna, *G*_*Rx*_ is the receiver antenna gain, and *c* is the speed of the light. Figure 18 depicts the obtained path loss in relation to the separation between the transmitting and receiving antennas. The obtained path loss is based on theoretical analysis; in real time, there will be many obstacles and losses in the signal.

The study of link budget is done to calculate the gain and loss of a signal in a system. It determines the reliability of signal transmission over different distances. The separation between the transmitter antenna and the receiver antenna is varied to determine signal reliability. The link margin can be calculated using Eq. (11)^26^.

11 $$Link\, margin=Antenna\, power \left({A}_{P}\right)-Required\, power \left({R}_{P}\right)$$

where antenna power (*A*_*p*_) can be calculated using Eq. (12), and the required power (*Rp*) can be computed using Eq. (13).

12 $${A}_{P}\left(dB\right)={P}_{Tx}+{G}_{Tx}+{G}_{Rx}-{L}_{F}-{P}_{L}$$

13 $${R}_{p}\left(dB\right)=\frac{{E}_{b}}{{N}_{0}}+K{T}_{0}+{B}_{r}$$

Here, transmission power is given by *P*_*Tx*_, transmitter gain is represented as *G*_*Tx*_, and gain of the receiver is denoted as *G*_*Rx*_, *L*_*F*_ is the free space loss, and *P*_*L*_ is the polarization mismatch loss. For 5 GHz and 28 GHz, the link margin is calculated using 0 dBm power as the input. At both the frequencies the gain of the transmitter is considered as 3 dBi^26,27^, while the gain of the receiver antenna is 3.95 dBi at 5 GHz and 7.5 dBi at 28 GHz.

It is determined that the polarization mismatch loss is 3 dB. When compared to optimal phase shift keying (PSK), the *E*_*b*_/*N*_0_ ratio is 9.6 dB. 203.93 dB/Hz is the noise power density (*N*_0_). *K* is the Boltzman’s constant, absolute zero temperature is given as *T*_0_, and *B*_*r*_ is the bit rate, which is 12 Mb/s. The given bit rate is sufficient to transfer a high-quality image. *L*_*F*_ can be expressed using Eq. (14).

14 $${L}_{F}=20{\log}\left(\frac{4\pi d}{\lambda}\right)$$

**Fig. 19:**
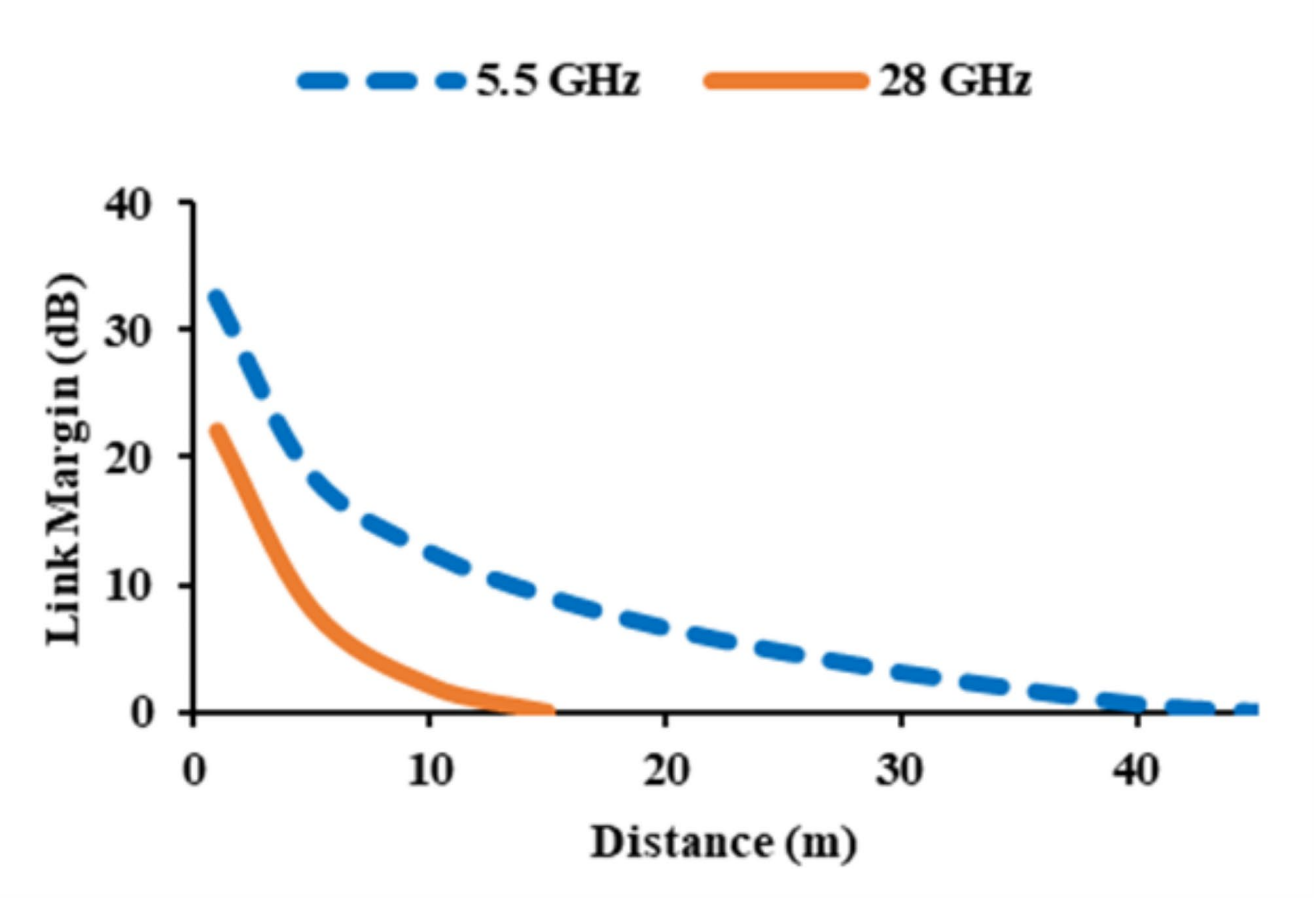
Link budget analysis.

Wavelength and distance *d* between the transmitter and receiver affects the value of *L*_*F*_. Figure 19 shows the data transmission range for both frequencies. If there is minimal cable loss, the data transmission range increases significantly. At 5.5 GHz, the data transmission range is 43 m, while at 28 GHz, the distance is 12.6 m.

#### Antenna housing

The SWB diverse antenna is a promising candidate for indoor wireless communication^5^. By increasing the bandwidth, one can combine multiple wireless technologies in one device to benefit the user. SWB antennas have a wide bandwidth, fast data transmission, lesser consumption of power, and minimal multipath fading. So, these antennas can be used in areas with high signal penetration, such as shopping malls, industries, hospitals, universities, airports, multistory buildings, and smart homes. Deep signal penetration is produced by channel path disruptions caused by obstacles such as doors, walls, objects, and celling^15^.

**Fig. 20:**
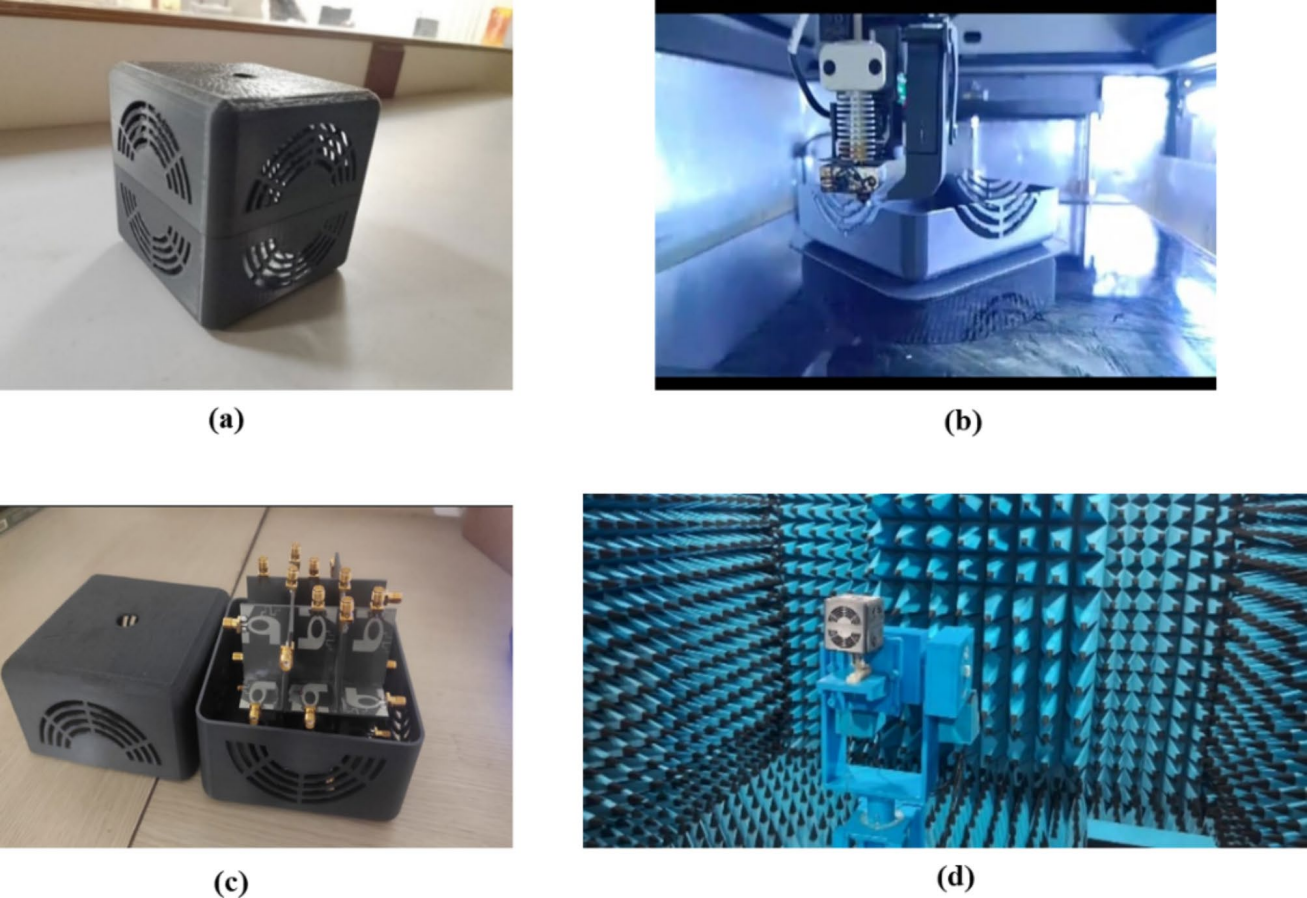
(**a**) Fabricated ABS casing, (**b**) RPT 3-D machine, (**c**) Antenna inside the casing, (**d**) Proposed antenna with ABS casing in an anechoic chamber.

**Fig. 21:**
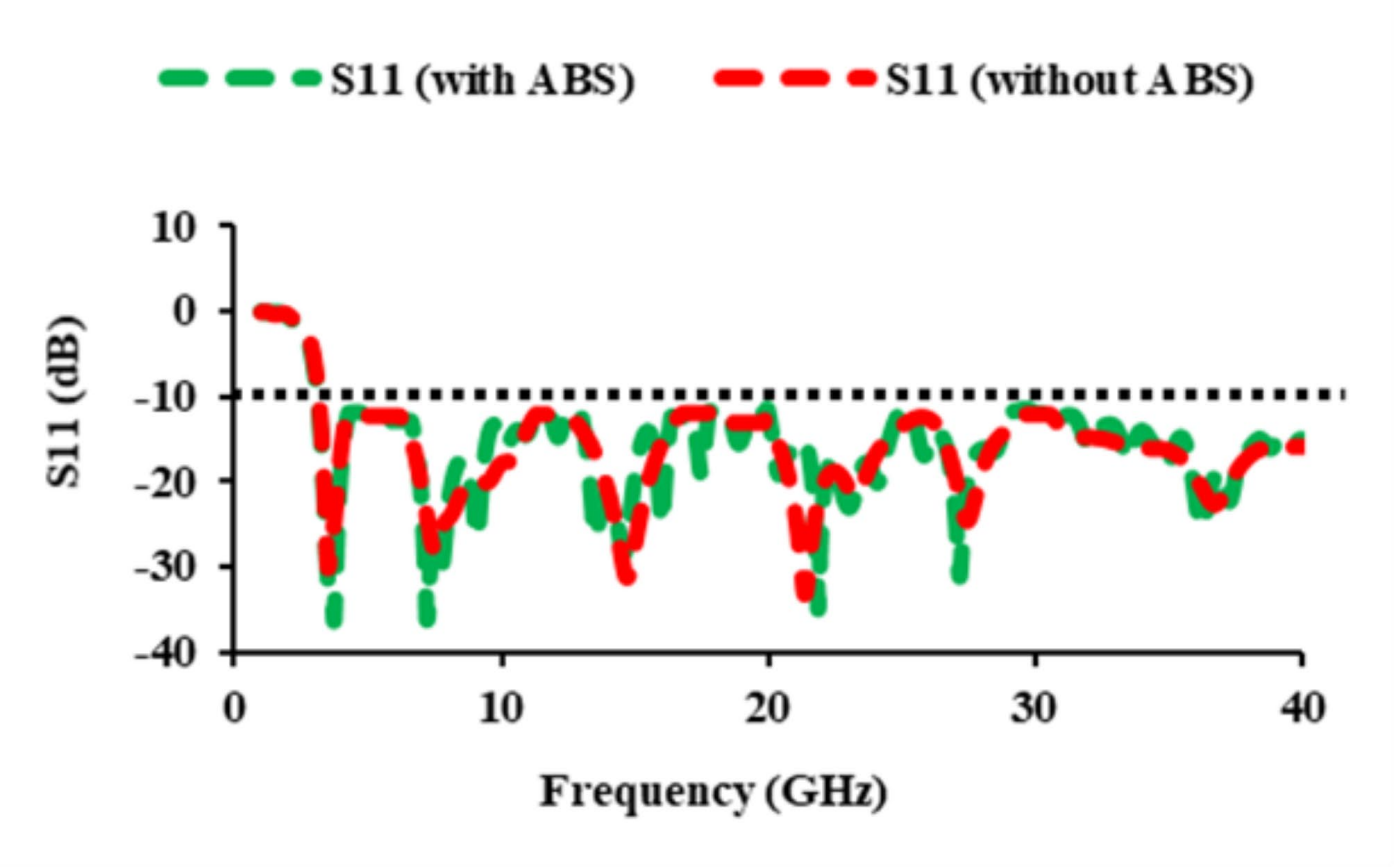
*S*_11_ of the designed antenna with and without ABS casing.

**Fig. 22:**
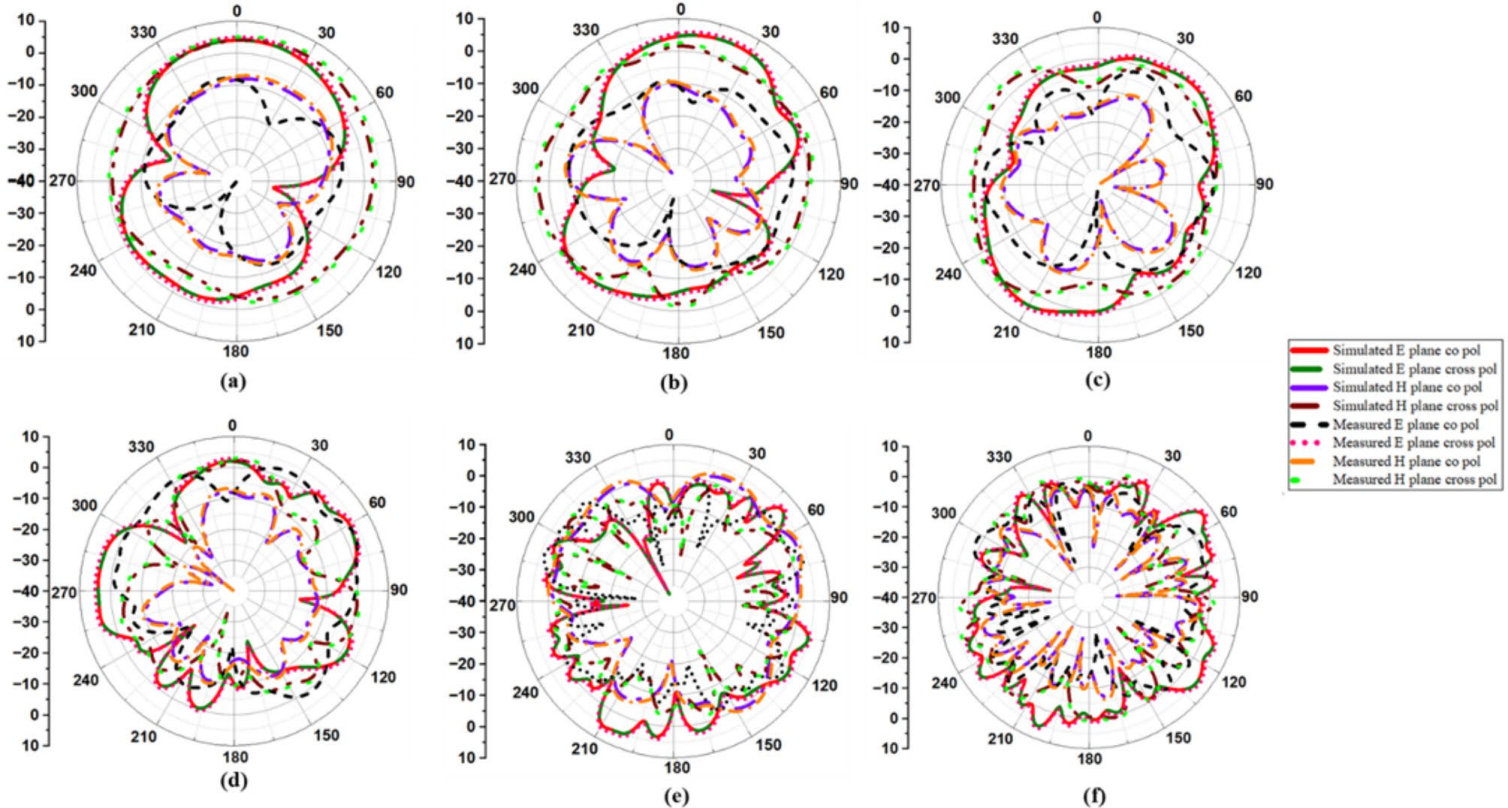
Co- and cross-polarization of the thirty-two port MIMO antenna with ABS casing: (**a**) 3.8 GHz, (**b**) 5.4 GHz, (**c**) 6.1 GHz, (**d**) 10.1 GHz, (**e**) 17.5 GHz, (**f**) 20.5 GHz.

An ABS casing is developed to test the antenna’s performance in the aforementioned real-time environments. ABS material has a dielectric constant of 2.8, so it is less sensitive to electromagnetic waves. The used ABS casing is square-shaped and best suited for mounting in building walls. Figure 20 depicts the ABS casing, its RPT 3-D machine for fabrication, the ABS with the fabricated thirty-two port SWB diverse antenna, and the measurement setup with the ABS casing in anechoic chamber. The *S*_11_ of the proposed antenna with and without ABS casing are displayed in Fig. 21, and it is found that even with and without ABS casing, the operating frequency is maintained between 3 and 40 GHz. Figure 22 depicts the radiation patterns of the constructed antenna with casing at 3.8 GHz, 5.4 GHz, 6.1 GHz, 10.1 GHz, 17.5 GHz, and 20.5 GHz frequencies, and it is evident that the even with casing radiation pattern is omnidirectional.

**Table 4:** Comparison of the developed SWB antenna to previously published SWB antennas in the literature.

Refs.	Unit cell dimensions (mm)	MIMO dimensions (mm)	Frequency (GHz)	Impedance BW (GHz)	No. of ports	BW ratio	Fractional BW (%)	Peak gain (dB)	Efficiency (%)	DG	Polarization vectors
^2^	29 × 22	58 × 58	2.9–40	37.1	4	13.79:1	–	13.5	–	> 9.5	Dual
^7^	32 × 32.5	63 × 63	0.97-35	34.03	4	36:1	–	5	–	–	Dual
^5^	30 × 20	60 × 55	2.3–23	20.7	4	10:1	–	4.5	90	> 9.94	Dual
^12^	31 × 31	34 × 60	2.3–18	15.7	2	7.83:1	–	6	–	> 9.99	Dual
^23^	49 × 35	116.5 × 84.5	2.5–20	17.5	4	6.90:1	–	5.2	–	–	Dual
^28^	20 × 20	68 × 68	3.1-28.78	25.68	8	9.28:1	–	–	87	> 9.9	Horizontal/ Vertical
^29^	95 × 80	–	7-34.6	27.6	2	4.94:1	132.69	9.5	–	> 9.95	Dual
^30^	–	90 × 45	2.2473-30	27.75	2	13.35:1	172.12	6.205	–	–	Single
^31^	20 × 20	–	3.6–36.5	32.9	2	10.14:1	–	6	–	> 9.98	Dual
^32^	18.3 × 15.9	41 × 41	1.9–20	18.1	4	10.53:1	–	8	89	> 9.98	Dual
^33^	–	26 × 15	3.1–35	31.9	2	11.29:1	–	–	> 50	> 9.9	Single
Prop.	20 × 22	82 × 82	3–40	37	32	13.33.1	172.09	12.5	94	> 9.9	Hex

Table 4 shows the similarities and dissimilarities of the constructed thirty-two SWB radiating antenna with existing SWB MIMO antenna designs. The table clearly shows that,

- The constructed antenna has a greater number of elements packed in a compact space than existing SWB antennas^2,5,7,12,23,29,30^.
- The previously reported antennas typically take up more space due to their larger single element design^5,7,12,23,28,30–33^, compared to the proposed antenna design.
- The obtained bandwidth ratio and FBW of the presented antenna are higher than^5,12,23,28,29,31–33^. Even though the antennas in^2,7^ have a higher bandwidth ratio and FBW, their single element size is larger than the unit cell dimension of the developed antenna.
- The developed antenna peak gain is higher and polarization vectors compared to previously reported antenna structures^5,7,12,23,29–32^. The antenna efficiency obtained is 94%, which is higher than antenna designs in the literature^5,28,33^.
- The literature suggests that the SWB MIMO antenna achieves greater diversity when arranged in 2-D rather than 3-D.
- The impact of antenna housing on various parameters, such as MEG, CCL, TARC, and ECC, has not been extensively studied in the literature.
- The maximum antenna elements used in SWB MIMO antennas is eight, according to the literature. The proposed antenna includes the greatest antenna elements. The polarisation vectors increase with the number of ports.

Thus, the proposed antenna achieves a wider impedance bandwidth while maintaining a compact arrangement. The number of elements placed takes up less space than other reported antennas. The achieved polarization vectors are higher, making the suggested antenna suitable for use in deep fading and large scattering environments. The antenna housing effect demonstrates that the characteristics of the antenna is not affected even when placed inside the casing. In addition, the proposed MIMO antenna can be used for massive MIMO technology by further increasing the number of antenna elements, thereby improving wireless network performance^34,35^. Massive MIMO systems can improve overall energy efficiency by spreading power over a large number of antennas^36^. In the future, the filtering mechanism can be combined with the proposed multi-port MIMO antenna to eliminate unwanted interfering frequencies from the SWB^37,38^.

## Conclusion

In this paper, a thirty-two port SWB diverse antenna is presented. The antenna has a compact structure and has an impedance bandwidth of 3 to 40 GHz. The MIMO elements are arranged in a 3-D orientation across five different planes, resulting in polarization diversity that improves link reliability. The obtained gain is 12.5 dBi and efficiency is 94%. The diverse properties of the developed antenna are found to be within the practical range. The antenna housing effect demonstrates that the antenna’s performance is unaffected even when placed inside a casing, allowing it to be used in environments with deep signal penetration. Therefore, the constructed antenna is appropriate for indoor wireless communication.

## Data Availability

The datasets used and analyzed during the current study are available from the corresponding author on reasonable request.
